# Structural and functional determinants of the archaeal 8-oxoguanine-DNA glycosylase AGOG for DNA damage recognition and processing

**DOI:** 10.1093/nar/gkac932

**Published:** 2022-10-27

**Authors:** Coste Franck, Goffinont Stéphane, Cros Julien, Gaudon Virginie, Guérin Martine, Garnier Norbert, Confalonieri Fabrice, Flament Didier, Suskiewicz Marcin Josef, Castaing Bertrand

**Affiliations:** Centre de Biophysique Moléculaire (CBM), UPR4301 CNRS, Université d’Orléans, CS 80054, rue Charles Sadron, F-45071 Orléans cedex 02, France; Centre de Biophysique Moléculaire (CBM), UPR4301 CNRS, Université d’Orléans, CS 80054, rue Charles Sadron, F-45071 Orléans cedex 02, France; Centre de Biophysique Moléculaire (CBM), UPR4301 CNRS, Université d’Orléans, CS 80054, rue Charles Sadron, F-45071 Orléans cedex 02, France; Centre de Biophysique Moléculaire (CBM), UPR4301 CNRS, Université d’Orléans, CS 80054, rue Charles Sadron, F-45071 Orléans cedex 02, France; Centre de Biophysique Moléculaire (CBM), UPR4301 CNRS, Université d’Orléans, CS 80054, rue Charles Sadron, F-45071 Orléans cedex 02, France; Centre de Biophysique Moléculaire (CBM), UPR4301 CNRS, Université d’Orléans, CS 80054, rue Charles Sadron, F-45071 Orléans cedex 02, France; Institut de Biologie Intégrative de la cellule (I2BC), UMR 9198 Université Paris-Saclay-CNRS-CEA, Bâtiment 21, Avenue de la Terrasse, F-91190 Gif-sur-Yvette, France; Université de Brest, Ifremer, CNRS, Unité Biologie et Ecologie des Ecosystèmes marins Profonds (BEEP), F-29280 Plouzané, France; Centre de Biophysique Moléculaire (CBM), UPR4301 CNRS, Université d’Orléans, CS 80054, rue Charles Sadron, F-45071 Orléans cedex 02, France; Centre de Biophysique Moléculaire (CBM), UPR4301 CNRS, Université d’Orléans, CS 80054, rue Charles Sadron, F-45071 Orléans cedex 02, France

## Abstract

8-Oxoguanine (GO) is a major purine oxidation product in DNA. Because of its highly mutagenic properties, GO absolutely must be eliminated from DNA. To do this, aerobic and anaerobic organisms from the three kingdoms of life have evolved repair mechanisms to prevent its deleterious effect on genetic integrity. The major way to remove GO is the base excision repair pathway, usually initiated by a GO-DNA glycosylase. First identified in bacteria (Fpg) and eukaryotes (OGG1), GO-DNA glycosylases were more recently identified in archaea (OGG2 and AGOG). AGOG is the less documented enzyme and its mode of damage recognition and removing remains to be clarified at the molecular and atomic levels. This study presents a complete structural characterisation of apo AGOGs from *Pyrococcus abyssi* (Pab) and *Thermococcus gammatolerans* (Tga) and the first structure of Pab-AGOG bound to lesion-containing single- or double-stranded DNA. By combining X-ray structure analysis, site directed mutagenesis and biochemistry experiments, we identified key amino acid residues of AGOGs responsible for the specific recognition of the lesion and the base opposite the lesion and for catalysis. Moreover, a unique binding mode of GO, involving double base flipping, never observed for any other DNA glycosylases, is revealed. In addition to unravelling the properties of AGOGs, our study, through comparative biochemical and structural analysis, offers new insights into the evolutionary plasticity of DNA glycosylases across all three kingdoms of life.

## INTRODUCTION

DNA of all living organisms is permanently subjected to chemical and physical stresses of endogenous or environmental origins capable of modifying its structure. When stress-induced structural changes lead to interference with DNA transactions (replication, recombination, transcription, etc.), we speak of DNA damage (1). In response to this constant risk, organisms have evolved various DNA repair systems which are conserved from prokaryotes to higher eukaryotes in terms of their general molecular strategies (2).

Reactive oxygen species (ROS) are responsible for numerous types of damage, such as the major oxidation product of purine in DNA and in the nucleotide triphosphate pool, the 8-oxoguanine (GO) and 8-oxodGTP (dGOTP) (3,4). GO (in DNA) and dGOTP (as a DNA polymerase substrate) are associated with error-prone replication, resulting in G to T and T to G tranversions, respectively (5–7). The mutagenic effect of GO is due to its peculiar base pairing properties. GO can form a base pair with C, GO:C, which is similar to the classical Watson–Crick G:C base pair. Alternatively, GO can form a Hoogsteen base pair with A, GO:A, which is stabilized by two hydrogen bonds and requires GO in *syn*-conformation and A in *anti*-conformation. As a result, most DNA polymerases indifferently incorporate C or A opposite GO. Both these processes lead to transversions.

To counteract GO-induced mutagenesis, organisms from all kingdoms of life possess a GO-specific repair system (8–11): (i) a GO-DNA glycosylase removes GO from DNA; (ii) an A/G specific-DNA glycosylase removes the normal adenine (A) misinserted by DNA polymerase opposite GO (12) and (iii) a dGOTPase sanitizes the nucleotide triphosphate pools by hydrolysing dGOTP to dGOMP and PPi (13). The first two of these activities initiate the Base Excision Repair (BER) pathway by cleaving the *N*-glycosidic bond between the base and the associated deoxyribose. The resultant abasic (AP) site is processed by an AP lyase (which can be present alongside the DNA glycosylase activity in one enzyme) and/or an AP endonuclease, and repair is terminated by a DNA polymerase and a DNA ligase (8). Although the GO-DNA glycosylase and the A/G specific-DNA glycosylase activities are not present in all species, the GO-repair system seems functional in most organisms studied to date (14–16).

Hyperthermophilic archaea are expected to be in a particular need of a functional GO-repair system, because ROS production is stimulated by high temperature, resulting in increased GO generation (17). While no enzymes with an A/G specific-DNA glycosylase or a dGOTPase activity have been discovered in these species, a GO-DNA glycosylase called AGOG (for Archaeal GO-DNA Glycosylase) has been initially identified in the hyperthermophilic archaeon *Pyrobaculum aerophilum* (18). Despite very low sequence identity, AGOG is structurally similar to eukaryotic and prokaryotic OGG1 and OGG2 GO-DNA glycosylases and, like these two proteins, belongs to the Endo III structural superfamily partly defined by the central helix-hairpin-helix (HhH) element followed by a glycine/proline-rich region and an aspartate (GPD). AGOG and OGGs are structurally distinct from—albeit functionally similar to—bacterial and eukaryotic GO-DNA glycosylases from the Fpg/Nei superfamily, which are characterised by a core helix-two turn-helix (H2TH) domain (9).

The AGOG enzymes that have been most studied to date include those from *Pyrobaculum aerophilum* (Pae-AGOG) (19,20), *Thermococcus gammatolerans* (Tga-AGOG) (21,22) and *Thermococcus kodakarensis* (Tko-AGOG) (23) archaea, all of which live at around 90–100°C. The crystal structure of Pae-AGOG in its free and 8-oxoguanosine (8-oxodG)-bound forms reveal a significant difference from human OGG1/2 in terms of the structure of the HhH-GPD motif and the mode of GO recognition (20). However, site-directed mutagenesis of K140 of the HhH motif and D172 of the GPD-peptide of Pae-AGOG demonstrates that these residues are functionally equivalent to K249 and D268 of hOGG1 and are absolutely necessary for enzyme catalysis, suggesting a conserved catalytic mechanism (19,24,25).

Despite the growing knowledge of AGOGs, its mode of interaction with damaged DNA remains to be elucidated. In this work, we present for the first time the X-ray 3D structures of Pab-AGOG and Tga-AGOG, as well as that of Pab-AGOG interacting with a short single- or double-stranded DNA fragment containing an AP site covalently linked to the catalytic lysine (K142). We also solved the structure of the inactive mutant K142Q-Pab-AGOG bound to a GO-containing double-stranded DNA duplex. By combining structural data with functional biochemistry experiments, we decipher at the atomic level the structural and functional determinants essential for AGOG-mediated damage recognition and catalysis.

## MATERIALS AND METHODS

### DNA probes

With the exception of the 24-mer F containing 2–6-diamino-4-hydroxy-5*N*-methylformamidopyrimidine (N7-meFapyG = F), which was generously donated to us by Professor Carmelo J. Rizzo (26), all synthetic single-stranded oligodeoxynucleotides (ODNs) used in this study were purchased from Eurogentec (Belgium) (Supplementary Figure S1). The lesion (X)-containing 24-mer strand (24-mer X) was 5’-[^32^P]-labelled. 5’-[^32^P]-24-mer X were used as substrates or ligands either directly or after its annealing with its cold complementary strand containing normal base (Y) opposite X to generate the 24-mer [X:Y] DNA duplex where X stands for 8-oxoguanine (GO), 8-oxoadenine (OA), uracil (U), abasic site (AP) or THF (tetrahydrofuran) AP site analogue and Y for C, G, A or T). The AP site-containing strands (24-mer AP) were obtained from the 24-mer U treated with uracil-DNA glycosylase as previously described (27). Cold GO-containing 5-, 7-, 9-, 11-, 13-mer single stranded ODN were used to explore the effect of the length of ODN on the ability of Pab-AGOG to form an imino-enzyme DNA intermediate (see DNA trapping assay and Supplementary Figure S1). A single- and double-stranded 57-mer containing GO or GO opposite C, respectively, was used to explore the effect of the incubation temperature on the Pab-AGOG activity (Supplementary Figures S1 and S2).

### Expression and purification of recombinant proteins

The recombinant *Lactococcus lactis* GO-DNA glycosylase (LlFpg) and human OGG1 (hOGG1) were produced and purified as previously described (27–29).

The wild type Pab-AGOG and Tga-AGOG ORFs amplified from Pab-1695-pGEX and Tg1653 were digested with NdeI and BamHI restriction enzymes and cloned into pET28a(+) vectors (Novagen). The expression plasmids, comprising N-terminal hexa-histidine-tagged (6HisTag) protein coding sequences, were transformed into *E. coli* Rosetta™ 2 (DE3) cells. The expression of the recombinant proteins was auto-induced (30) and after 16 h at 20°C, cells were harvested by centrifugation (4000 g for 1 h at 4°C) and pellets were stored at –80°C. Cell pellets were resuspended in lysis Buffer (Buffer 1:20 mM HEPES pH 7.6, 500 mM NaCl and 5 mM imidazole) and lysed by freeze-thaw/sonication in the presence of lysozyme (0.7 mg/ml, Sigma-Aldrich). The cell lysate fraction containing soluble proteins was clarified by centrifugation (19 000 g for 60 min at 4°C). The supernatants were incubated at 80°C for 10 min, centrifuged (19 000 g for 45 min at 4°C) and then filtered (0.45 μm) to get rid of the unfolded protein contaminants. Soluble protein fractions were collected and applied onto a 5 ml Co^2+^-Talon resin (Clontech) for immobilized metal-ion-affinity chromatography (IMAC) (Sigma-Aldrich) equilibrated in Buffer 1. The N-terminal 6HisTag-AGOG enriched IMAC fraction eluted with Buffer 2 (20 mM HEPES pH 7.6, 500 mM NaCl and 20 mM imidazole) was diluted 3 times with Buffer 3 (20 mM HEPES pH 7.6) prior to be applied to a POROS™ HS20 cation exchanger column (Applied Biosystems). Proteins were eluted using a 40 CV linear salt gradient from 0.1 to 1 M NaCl in Buffer 3. The pooled 6HisTag-AGOG-containing fractions were concentrated using Ultracel-10 units (Millipore) prior to Tag cleavage by trypsin, as thrombin was shown to be ineffective. To remove uncleaved fusion proteins and the trypsin protease, incubation mixture was applied onto a 1 ml HiTrap benzamidine FF column (GE Healthcare) on top of the cobalt affinity column and the flowthrough was concentrated using Ultracel-10 units. For the last purification step, the AGOG proteins were loaded onto a Superdex 75 column (GE Healthcare) equilibrated in Buffer 4 (20 mM HEPES pH 7.6, 1 M NaCl). The homogeneous protein Pab-AGOG was finally concentrated by ultrafiltration (Ultracel-10 units) to 34 mg/ml and stored in Buffer 5 (20 mM HEPES pH 7.6, 250 mM NaCl, 1 mM TCEP) whereas Tga-AGOG was stored at 20 mg/ml in Buffer 6 (20 mM HEPES pH 7.6 and 750 mM NaCl). Purified proteins were analysed by standard SDS-PAGE and their molecular weights were determined by MALDI-TOF MS. For Pab-AGOG, the observed mass (27 943 Da) was in agreement with a trypsin cleavage at the thrombine site (27 944 Da). For Tga-AGOG, the observed mass (29 671 Da) was in agreement with a protein cleavage site between R8 and I9 (29 674 Da).

### GO excision and AP site cleavage enzyme assays

For DNA glycosylase/AP lyase and AP lyase activities, lesion-containing strand was labelled at its 5' terminus with γ-[^32^P]-ATP and annealed (or not, for a single-stranded substrate) with the cognate complementary strand. Assays (20 μl) were performed at 37°C and at concentrations of 20 nM for DNA, 2 nM for enzyme under multi-turnover (MTO) conditions or 200 nM for single-turnover condition (STO). A control time course with DNA alone to test its stability during incubation is performed and subtracted from the time course with enzyme if necessary. The reactions were stopped by addition of 1 volume of 20 mM NaOH and incubation for 2 min at room temperature before addition of formamide–SDS loading buffer (75% formamide, 1 mM EDTA, 0.2% SDS, final concentrations) and denatured at 50°C for 3 min just before loading on an urea-denaturing 20% polyacrylamide gel (urea–PAGE). For glycosylase activity, reactions were stopped by adding 1 volume of 0.4 M NaOH and incubation for 2 min at 50°C before the addition of formamide-SDS loading buffer. Substrate and product were separated by electrophoresis (20 min at 50 mA/gel—Protean III, Bio-Rad). The bands were visualized on a TyphoonTM Fluoroimager (GE Healthcare) and quantified using ImageQuant TL (GE Healthcare). The cleavage data from at least three independent experiments was analysed using OriginLab® software. The rate constants were calculated by fitting the cleavage data to the BoxLucas1 exponential function, *y* = *a*(1 – exp(–*b*× *x*)), where *b* is the single turnover rate constant (*k*_obs_) and *x* the time (Supplementary Figure S3).

### Electrophoretic mobility shift assay (EMSA)

DNA binding properties of AGOG variants used in this study were analysed by electrophoretic mobility shift assay (EMSA) using the general procedure already described (31). Briefly, 0.1 nM of 5’-[^32^P]-labeled 24-mer single- [X] or double-stranded DNA [X:Y] (where X = GO or THF, Supplementary Figure S1) was incubated at 4°C for 30 min alone or with the indicated protein concentration in 20 mM HEPES/NaOH, pH 7.6, 100 mM NaCl, 10% glycerol, 0.1% BSA. Binding reactions were loaded onto a non-denaturing 10% polyacrylamide gel for electrophoresis as described (32). After electrophoresis (14 V/cm at 4°C), dried gels were exposed for autoradiography using a Typhoon Molecular Imager and quantified using ImageQuant software (Supplementary Figure S9). Triplicate EMSA titration experiments were used to extract apparent dissociation constants *K*_D__app_ which is close to the enzyme concentration needed for half-maximal binding under the experimental conditions chosen (32). The binding points were fitted using a non-linear regression logistics function (Hill's equation, *Y* = *Y*_max_ × *X_n_*/(*K*_D__*n*_ + *Xn*) with Ymax corresponding to the protein maximal DNA binding, *X* the protein concentration in the assay, *K*_D_ the dissociation constant, i.e. the concentration of protein for half-maximal binding and, n the Hill's coefficient) with software Origins, version 9.0.0 (OriginLab, Northampton, MA, USA) (Supplementary Figure S4).

### Trapping assay

The Schiff base (SB) covalent intermediate between the bifunctional DNA glycosylases Pab-AGOG, hOGG1 or LlFpg and single- or double-stranded 24-mer DNA was trapped by its irreversible reduction (reduced Schiff base, rSB) with 0.1 M sodium borohydride (NaBH_4_) as follows. Reaction mixtures containing 20 nM of single- or double-stranded DNA radiolabelled on the GO- or AP site-containing strand and 0.1 M NaBH_4_ were incubated alone or with indicated protein for 15 min at 37°C in 10 mM TE 1×, pH 7.5, 150 mM NaCl, 1 mM EDTA and 0.1 mg/ml BSA. Reactions were stopped by the formamide-SDS loading buffer and analysed by 20% urea–PAGE. Gels were then exposed for autoradiography using a TyphoonTM Fluoroimager.

Trapping assays were also performed with cold GO-containing single-stranded DNA substrates (5-mer to 13-mer, Supplementary Figure S1) and Pab-AGOG to evaluate the effect of the DNA length on the trapping reaction and to optimise the homogeneity of rSB for crystallization. For that, Pab-AGOG (300 μM final concentration) was added to a mixture containing single-stranded DNA in 1.5–2 molar excess and 0.1 M NaBH_4_ in Buffer 6 (20 mM HEPES/NaOH, pH 7.5 and 350 mM NaCl), and incubated at 20°C for 10–30 min. When required, reactions were stopped by the addition of the Laemmli loading buffer and the resulting mixtures were analysed by SDS-PAGE on 10% EZ-run gels (Fisher Scientific).

### Preparation of the borohydride-trapped DNA-AGOG covalent complexes for crystallization

Trapping reaction was performed as described above with cold GO-containing single-strand DNA. The purification of the borohydride-trapped Pab-AGOG-DNA complex (rSB) is based on several chromatographic steps: a size-exclusion chromatography run at high ionic strength on a HiLoad Superdex 75™ (S75) column followed by several cation exchanger chromatography on Mono Q 5/50 GL column. During purification, the homogeneity of the elution fractions was analysed by SDS-PAGE. The trapping reaction was stopped in ice by the addition of NaCl to a final concentration of 1 M. The high ionic strength allows stopping the reaction and dissociating the remaining non-covalent Pab-AGOG-DNA complexes that were not trapped by NaBH_4_ (enzyme/substrate or enzyme/product). Briefly, the trapping-reaction mixture was loaded onto Superdex 75 column equilibrated in Buffer 6 containing 1 M NaCl (S75 fraction). The size exclusion chromatography step allowed us to eliminate the non-trapped DNA that was present in excess in the trapping reaction. After its 10-fold dilution with buffer 6 without NaCl, the S75 fraction was loaded onto the Mono Q 5/50 GL column equilibrated in buffer 6 without NaCl. rSB was eluted by a linear 0.05–1 M NaCl gradient at around 150 mM NaCl. Several runs of Mono Q loading/elution were required to purify to homogeneity the trapped product rSB. rSB corresponding to the trapped complex Pab-AGOG-9-mer single-stranded DNA (PabAGOG-ssDNA) was then concentrated in buffer 6–9 mg/ml and stored at 4°C. To prepare trapped complex of Pab-AGOG bound to double-stranded DNA (Pab-AGOG-dsDNA-Y), the trapped complex of Pab-AGOG-ssDNA was incubated for 5 min at 60°C with 1.2 molar excess of its complementary 9-mer Y complementary stranded DNA (with Y = C, T, G or A, Supplementary Figure S1) and annealed by slowly lowering the temperature to 4°C to generate [Pab-AGOG-dsDNA-C, -T, -G or –A] with a DNA duplex harbouring the lesion opposite C, T, G or A, respectively.

### Crystallization and X-ray structure determination

Crystallization condition screening was performed using kits from Molecular Dimensions Ltd (JCSG Plus, Morpheus, Wizard Classic, Structure screen, and—only for complexes—HELIX) *via* the sitting-drop vapour-diffusion method using a Mosquito® liquid handler instrument (TTP LabTech). Well diffracting crystals of Pab-AGOG were obtained at 20°C in JCSG Plus condition 26 (1 M LiCl, 100 mM tri-sodium citrate pH 4.0 and 20% PEG 6000) with the protein concentrated to 32 mg/ml. After 2 weeks, crystals were transferred into mother liquor supplemented with 25% ethylene glycol and then flash-frozen in liquid nitrogen. Crystals of Pab-AGOG complexed with 8-hydroxy-2’-deoxyguanosine (8oxo-dG) [Pab-AGOG + 8oxodG] were prepared by adding small amount of the 8oxo-dG powder (Sigma-Aldrich, H5653) to native crystal droplets for several days. Crystals were flash-frozen like the native crystals. Tga-AGOG at 20 mg/mL crystallized in JCSG Plus condition 37 (24% PEG 1500, 20% glycerol) at 20°C. Flash-frozen crystals were obtained without addition of a cryoprotectant.

Crystals of single-stranded DNA trapped to the Pab-AGOG protein [Pab-AGOG_ssDNA] were obtained at 20°C in the Helix 1.2 condition (1 mM spermine, 50 mM MES pH 6.5, 25% PEG 400) and were flash-frozen in mother liquor.

For the three complexes with a double-stranded DNA trapped to Pab-AGOG [Pab-AGOG_dsDNA-C, Pab-AGOG_dsDNA-T, Pab-AGOG_dsDNA-A], the best diffracting crystals appeared in the Helix 2.28 condition (10 mM MgCl_2_, 50 mM sodium acetate pH 5.0, 21% MPD) at 4°C.

To obtained crystals of Pab-AGOG non-covalently bound to DNA containing GO opposite C, DNA duplex was first separately prepared by annealing 9-mer GO with 9-mer C (Supplementary Figure S1). Thus, the DNA-protein complex was obtained by incubating for 30 min at 4°C the 9-mer GO:C duplex in a 1.2 molar excess with the catalytically defective mutant K142Q Pab-AGOG before starting crystallization screening. Crystals of K142Q Pab-AGOG/9-mer GO:C complex [Pab-AGOG/dsDNA-GOC] were obtained at 4°C in the Helix 2.27 condition (150 mM KCl, 50 mM sodium acetate pH 4.5, 32% MPD) and directly flash-frozen in liquid nitrogen.

100 K X-ray data were collected at SOLEIL synchrotron on PROXIMA-1 or PROXIMA-2 beamlines and processed using XDS (33) and AIMLESS (34) or autoPROC (35). Crystal structures were determined by molecular replacement using Phaser (36) of the Phenix suite (37). Atomic models were refined using phenix.refine and manually improved using COOT (38). Data collection and refinement statistics are listed in Table 1. Stereochemical validation of the final models were performed with Molprobity (39) and molecular graphics images were produced using UCSF Chimera (40).

**Table 1.** X-ray data collection and refinement statistics

**Data collection statistics**								
	Pab AGOG	Pab AGOG with 8oxo-dG	Trapped Pab AGOG on SS DNA	Trapped Pab AGOG on DS DNA-C	Trapped Pab AGOG on DS DNA-T	Trapped Pab AGOG on DS DNA-A	Pab AGOG on DS DNA-C	Tga AGOG
Radiation source	SOLEIL PROXIMA 1	SOLEIL PROXIMA 1	SOLEIL PROXIMA 2	SOLEIL PROXIMA 1	SOLEIL PROXIMA 1	SOLEIL PROXIMA 2	SOLEIL PROXIMA 2	SOLEIL PROXIMA 1
Wavelength (Å)	0.97856	0.97857	0.98011	0.97856	0.97856	0.98011	0.98011	0.97857
Spacegroup	*P*1	*C*2	*P*1	*C*222_1_	*C*222_1_	*C*2	*C*222_1_	*P*2_1_2_1_2_1_
cell dimensions: *a, b, c* (Å)	35.57, 35.92, 46.49	131.06, 47.20, 91.27	39.74, 74.26, 101.73	59.60, 71.83, 138.54	59.78, 71.56, 138.69	61.85, 68.78, 68.42	62.26, 68.81, 140.39	61.43, 62.45, 139.88
*α, β, γ* (°)	95.50, 91.89, 108.41	90.00, 100.80, 90.00	92.48, 100.74, 105.46	90.00, 90.00, 90.00	90.00, 90.00, 90.00	90.00, 90.35, 90.00	90.00, 90.00, 90.00	90.00, 90.00, 90.00
Number of molecules/ asymmetric unit	1	2	4	1	1	1	1	2
Resolution range (Å)	46.17–1.10 (1.12–1.10)	48.19–1.65 (1.74–1.65)	99.46–2.04 (2.08–2.04)	46.18–1.70 (1.73–1.70)	45.88–1.12 (1.14–1.12)	68.42–1.33 (1.35–1.33)	46.17–1.25 (1.27–1.25)	69.94–1.49 (1.52–1.49)
Total observations	593 573 (27 417)	212 816 (31 973)	497 667 (23 314)	295 391 (10 787)	1 435 524 (34 187)	430 766 (16 950)	1 085 592 (39 840)	645 645 (31 495)
Unique reflections	84 275 (4021)	65 858 (9640)	68 577 (3476)	33 127 (1726)	114 280 (5266)	66 075 (3311)	83 399 (4101)	88 713 (4393)
Completeness (%)	95.7 (92.0)	99.3 (99.9)	98.1 (96.9)	99.9 (99.3)	99.4 (91.7)	99.5 (100.0)	99.9 (99.9)	100.0 (100.0)
Multiplicity	7.0 (6.8)	3.2 (3.3)	7.3 (6.7)	8.9 (6.2)	12.6 (6.5)	6.5 (5.1)	13.0 (9.7)	7.3 (7.2)
*R*_merge_^a^ (%)	6.9 (37.4)	8.1 (40.4)	11.8 (88.4)	8.8 (97.8)	6.4 (59.8)	4.4 (49.8)	4.6 (35.4)	7.1 (84.4)
Average *I/*σ*(I)*	14.5 (4.4)	7.3 (2.3)	11.4 (2.1)	12.0 (1.5)	17.7 (2.3)	17.2 (2.2)	28.5 (5.3)	13.6 (2.3)
CC_1/2_ (%)	99.6 (95.6)	99.2 (83.7)	99.7 (76.6)	99.8 (72.2)	99.9 (83.4)	99.9 (92.4)	99.9 (95.8)	99.8 (83.2)
**Refinement and model statistics**								
Resolution range (Å)	46.17–1.10	44.83–1.65	71.23–2.04	45.87–1.70	34.65–1.12	45.99–1.33	46.17–1.25	46.15–1.49
Number of reflections used	84269	65723	68551	33030	114270	66042	83396	88699
*R*_work_^b^ */ R*_free_^c^ (%)	14.37/15.72	16.1/19.1	17.6/22.3	16.8/20.6	12.5/14.3	17.5/18.7	13.6/16.1	17.3/19.2
Average *B* values (Å^2^)								
All atoms	18.81	31.90	33.48	32.89	18.14	29.16	20.95	31.09
Protein chain A atoms	17.89	34.15	25.33	29.10	15.66	25.77	16.57	30.70
Protein chain B atoms	-	28.36	-	-	-	-	-	30.87
Protein chain C atoms	-	-	27.25	-	-	-	-	-
Protein chain E atoms	-	-	36.57	-	-	-	-	-
Protein chain G atoms	-	-	37.06	-	-	-	-	-
DNA chain B atoms	-	-	42.89	47.36	22.95	38.30	28.99	-
DNA chain D atoms	-	-	44.39	-	-	-	-	-
DNA chain F atoms	-	-	64.32	-	-	-	-	-
DNA chain H atoms	-	-	65.10	-	-	-	-	-
DNA chain I atoms	-	-	-	51.23	27.39	43.16	41.76	-
2’-Deoxy-8-oxoguanosine atoms	-	20.46	-	-	-	-	-	-
Ethane-1,2-diol atoms	39.33	39.01	-	-	-	-	-	-
Citric acid atoms	19.11	-	-	-	-	-	-	-
Sodium atoms	20.66	-	-	-	18.61	-	-	-
Potassium atoms	-	-	44.20	-	-	-	-	-
Phosphate atoms	-	-	56.73	-	-	-	-	-
Chloride atoms	21.29	-	-	-	-	-	-	19.28
Glycerol atoms	-	-	-	-	-	-	-	33.04
Methyl-pentanediol	-	-	-	-	16.66	21.55	36.8	-
Water atoms	30.48	40.39	36.37	39.16	33.44	37.36	32.51	34.86
Root mean square deviation from ideality								
Bond lengths (Å)	0.008	0.013	0.007	0.018	0.007	0.005	0.006	0.006
Bond angles (°)	1.007	1.140	0.793	1.557	1.014	0.823	0.904	0.766
Ramachandran analysis								
Favoured regions/ allowed regions/ outliers (% of residues)	98.3/1.7/0.0	99.2/0.8/0.0	97.7/2.3/0.0	98.3/1.7/0.0	98.8/1.2/0.0	98.3/1.7/0.0	99.2/0.8/0.0	97.9/2.1/0.0
Number of atoms								
Protein chain A	4053	1943	1932	1994	4099	1971	1997	1986
Protein chain B	-	1948	-	-	-	-	-	1918
Protein chain C	-	-	1924	-	-	-	-	-
Protein chain E	-	-	1900	-	-	-	-	-
Protein chain G	-	-	1876	-	-	-	-	-
DNA chain B	-	-	161	167	273	167	179	-
DNA chain D	-	-	161	-	-	-	-	-
DNA chain F	-	-	167	-	-	-	-	-
DNA chain H	-	-	140	-	-	-	-	-
DNA chain I	-	-	-	185	288	187	185	-
2’-Deoxy-8-oxoguanosine	-	40	-	-	-	-	-	-
Ethane-1,2-diol	70	20	-	-	-	-	-	-
Citric acid	18	-	-	-	-	-	-	-
Sodium	2	-	-	-	2	-	-	-
Potassium	-	-	1	-	-	-	-	-
Phosphate	-	-	10	-	-	-	-	-
Chloride	1	-	-	-	-	-	-	2
Glycerol	-	-	-	-	-	-	-	12
Methyl-pentanediol	-	-	-	-	44	8	16	-
Water	196	336	721	280	409	317	305	319
**PDB code**	7OLB	7OLI	7OUE	7OY7	7P0W	7P9Z	7P8L	7OU3

^a^ ${R_{{\rm{merge}}}} = \sum\nolimits_h {\sum\nolimits_i {| {{I_{h,i}} - {{\langle I \rangle }_h}} |} } /\sum\nolimits_h {\sum\nolimits_i {{I_{h,i}}} }$ where <*I>_h_* is the mean intensity of the symmetry-equivalent reflections.

^b^ ${R_{{\rm{work}}}} = \sum\nolimits_h {| {| {{F_o}} | - | {{F_c}} |} |} /\sum\nolimits_h {| {{F_o}} |}$ , where *F_o_* and *F_c_* are the observed and calculated structure factor amplitudes, respectively, for reflection h.

^c^ *R*_free_ is the *R* value for a subset of 5% of the reflection data, which were not included in the crystallographic refinement.

## RESULTS AND DISCUSSION

### Functional and structural characterization of AGOG from *Pyrococcus abyssi* and *Thermococcus gammatolerans*

**Figure 1.**
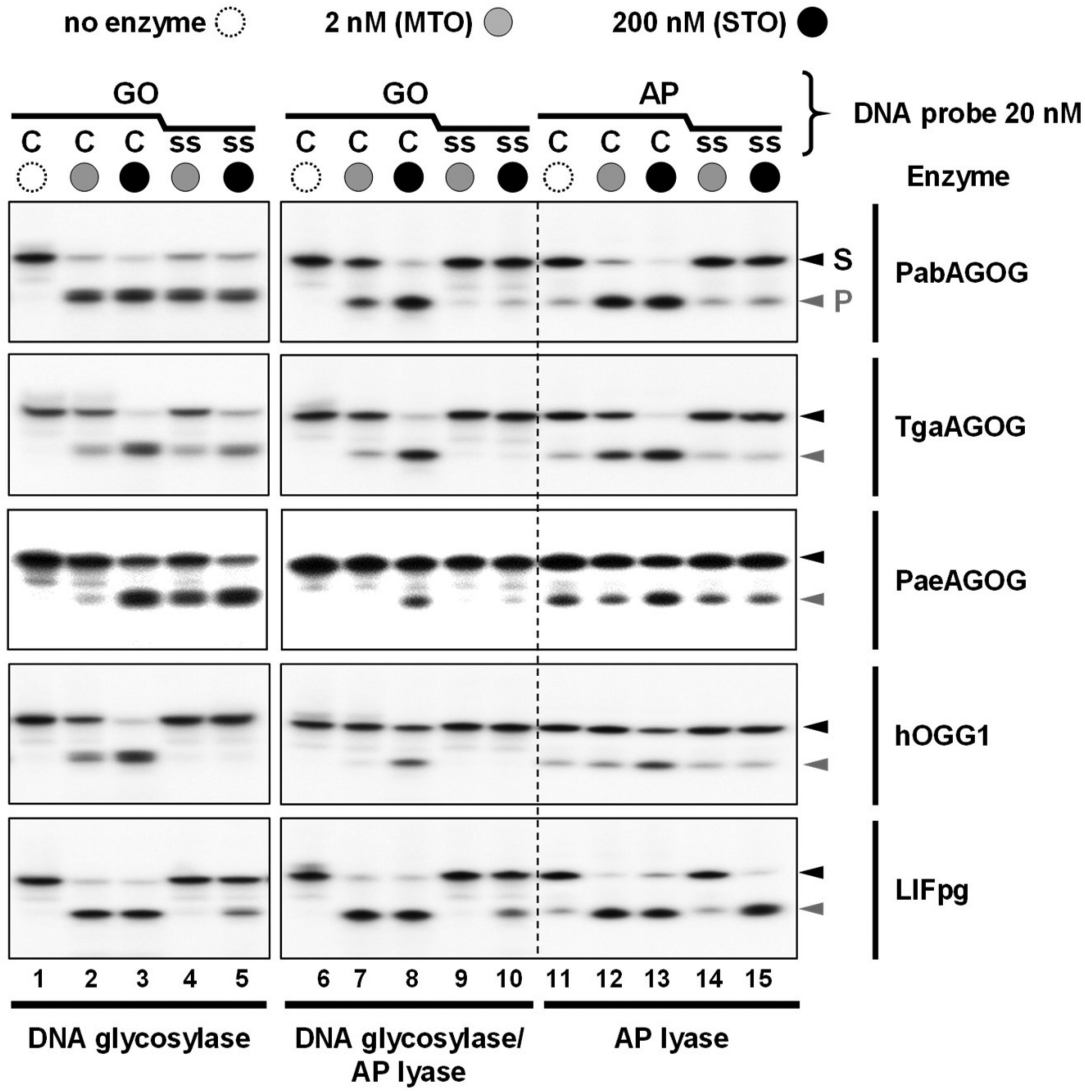
Comparative enzyme activities of archaeal, bacterial and eukaryotic GO-DNA glycosylases. As indicated, 5’-[^32^P]-labeled GO- (or AP site)-containing single (ss) or double-stranded (GO:C or AP:C) 24-mer DNA duplex was incubated for 15 min at 37°C alone (lanes 1, 6 and 11), with 2 nM (lanes 2, 4, 7, 9, 12 and 14) or with 200 nM (lanes 3, 5, 8, 10, 13 and 15) of indicated enzyme. Reaction mixtures were analyzed by urea–PAGE as described in Materials and Methods. Representative gel autoradiographs are shown. S and P are for DNA substrate and cleavage end-product, respectively. MTO, STO, Fpg, OGG1, Pab, Tga, Pae, h and Ll are defined in abbreviation list.

AGOGs from Pab, Tga and Pae were purified to homogeneity and their GO-DNA glycosylase, concerted GO-DNA glycosylase/AP lyase and AP lyase activities evaluated on single- and double-stranded DNA and compared to those of the GO-DNA glycosylases Fpg from *Lactococcus lactis* (LlFpg) and human OGG1 (hOGG1) (Figure 1). As expected and similarly to Pae-AGOG and Tga-AGOG, Pab-AGOG is a true GO-DNA glycosylase on both double-stranded (lanes 2 and 3) and single-stranded (lanes 4 and 5) DNA. Moreover, unlike Tga-AGOG, Pae-AGOG, and hOGG1, but similarly to Fpg, Pab-AGOG displays a good DNA glycosylase turnover on both single- and double-stranded DNA (compare multi-turnover conditions, MTO, i.e. [DNA/protein] molar ratio of 10 in lanes 2 and 4 with single-turnover conditions STO, i.e. [DNA/protein] molar ratio of 0.1 in lanes 3 and 5, respectively, Figure 1). Regarding the concerted GO-DNA glycosylase/AP lyase activity on double-stranded DNA, only Fpg and AGOGs are efficient bifunctional DNA glycosylases (lanes 7 and 8), but the turnover of Fpg is much higher than that of AGOGs at 37°C, as reflected in all of the generated AP sites being cleaved to the DNA end product by Fpg but not AGOGs under MTO conditions (lane 7). The last observation must be weighed against the fact that the comparison between Fpg and AGOGs cannot be performed under the physiological temperature conditions for hyperthermophilic enzymes. However, we performed an additional experiment to show that relative trends observed at 37°C hold also at higher temperatures (Supplementary Figure S2). As expected and similarly to Pae- and Tga-AGOG, Pab-AGOG is stimulated by a rise in temperature. Whatever the incubation temperature, Pab-AGOG is more effective on double-stranded DNA than on single-stranded DNA. Under the temperature range considered, GO-DNA glycosylase appears to be moderately and significantly decoupled from AP lyase with dsGO:C and ssGO, respectively. As was already known from previous work, hOGG1 is not a true bifunctional enzyme (compare lanes 2 and 3 with lanes 7 and 8, respectively, Figure 1), because the free GO base may remain in the enzyme active site after the glycosylase process thus contributing to the uncoupling of the two activities (41,42). Among the enzymes studied, only Fpg displays a true bifunctional activity on GO-containing single-stranded DNA, albeit with a much lower turnover than on the double-stranded DNA (compare lanes 7 and 8 with lanes 9 and 10, respectively). Using AP site-containing DNA as a substrate, it appears that AGOGs, like Fpg but in contrast to hOGG1, display an efficient AP lyase activity on double-stranded DNA (lanes 12 and 13). However, only the bacterial Fpg enzyme is able to efficiently cleave AP site-containing single-stranded DNA (again with a lower turnover than for double-stranded DNA—compare lanes 14 and 15 with 12 and 13, respectively). The most remarkable result in these experiments is the observation that, like hOGG1, all three AGOGs are unable to cleave the AP site in single-stranded DNA neither through the coupled GO-DNA glycosylase/AP lyase nor the AP lyase process.

**Figure 2.**
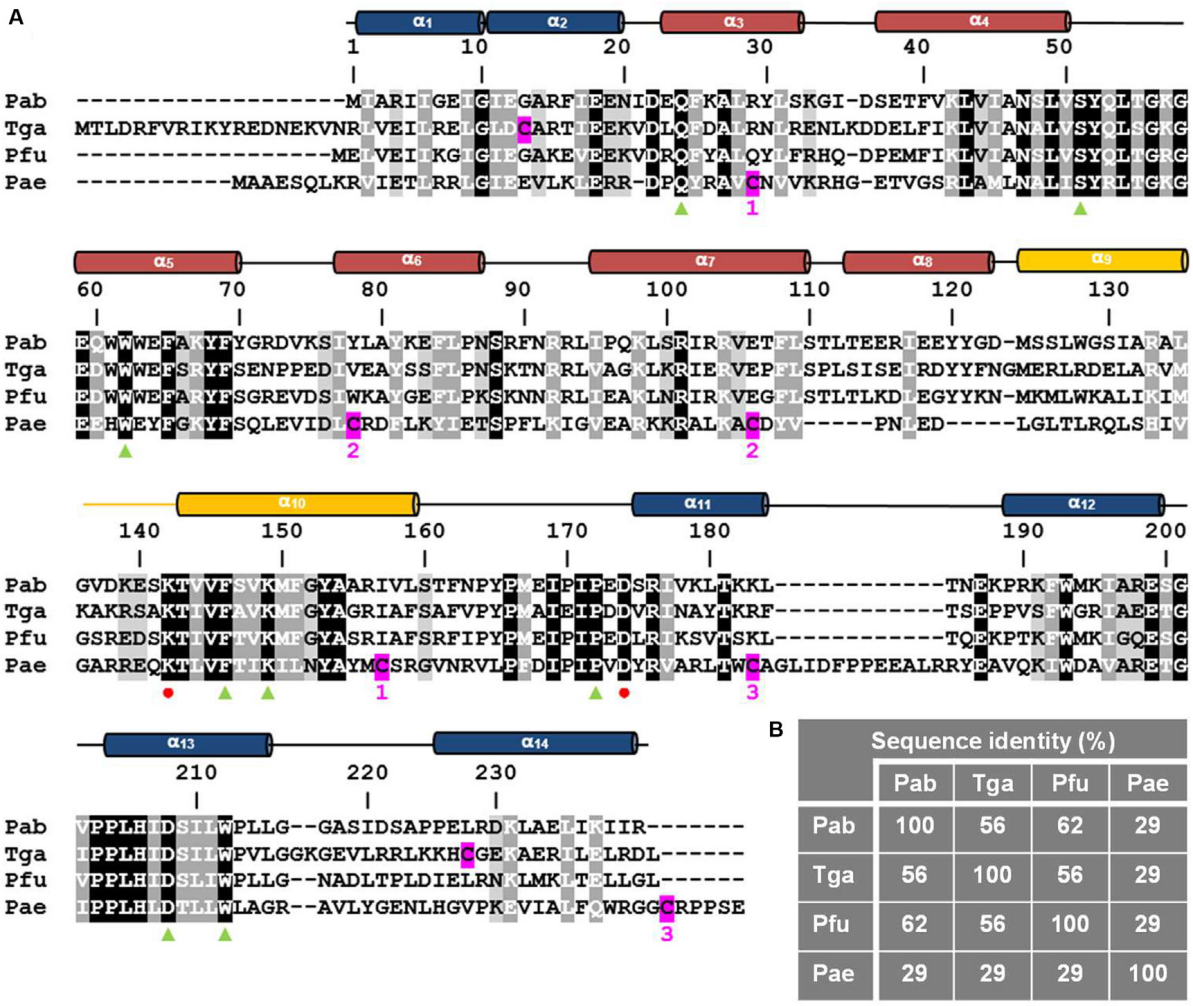
Primary and secondary structures of AGOGs. (**A**) Sequence alignment of AGOG proteins with known 3D structures. Secondary structures are colored according to Lingaraju *et al.* (*N/C* domain in *blue*, *HhH* motif in *yellow* and HhH domain in *red*) (20). *Red* dots indicate the positions of the catalytic residues K142 and D174 and *green* triangles indicate the position of the 8-oxodG-interacting residues. Cysteine residues are highlighted in *magenta*. (**B**) Sequence identity between Pab-AGOG and Tga-AGOG, Pfu-AGOG or Pae-AGOG.

**Figure 3.**
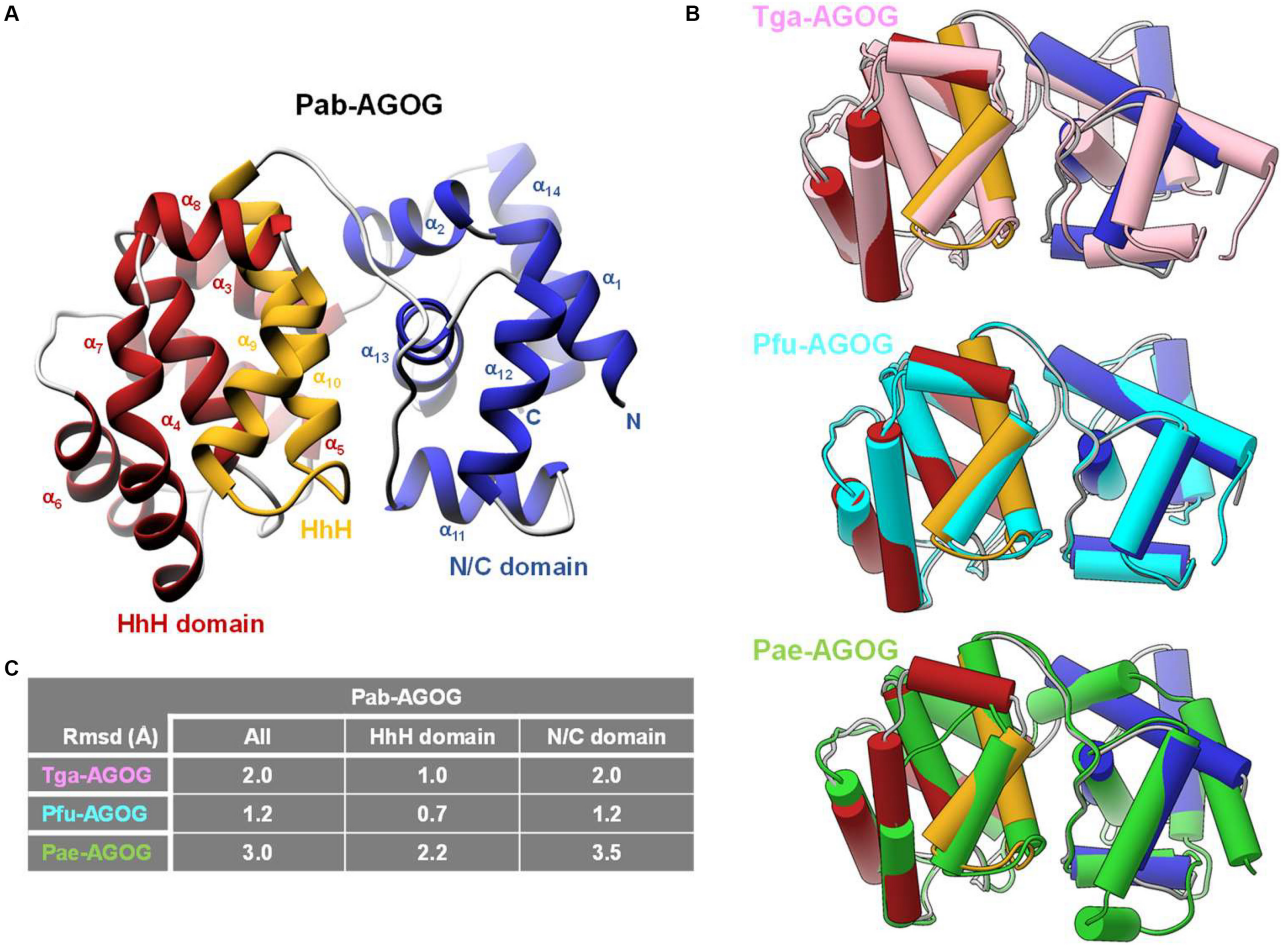
apo AGOG 3D structures. (**A**) Ribbon overall 3D structure of Pab-AGOG. The *N/C* domain is shown in *blue*, the HhH domain is shown in *red* and the HhH motif is highlighted in *yellow*. (**B**) Superposition of Pab-AGOG with Tga-AGOG (*pink*), Pfu-AGOG (*cyan*) or Pae-AGOG (*green*). (**C**) Rmsd values between Cα atoms of Pab-AGOG and Tga-AGOG, Pfu-AGOG or Pae-AGOG calculated using Matchmaker of ChimeraX.

**Figure 4.**
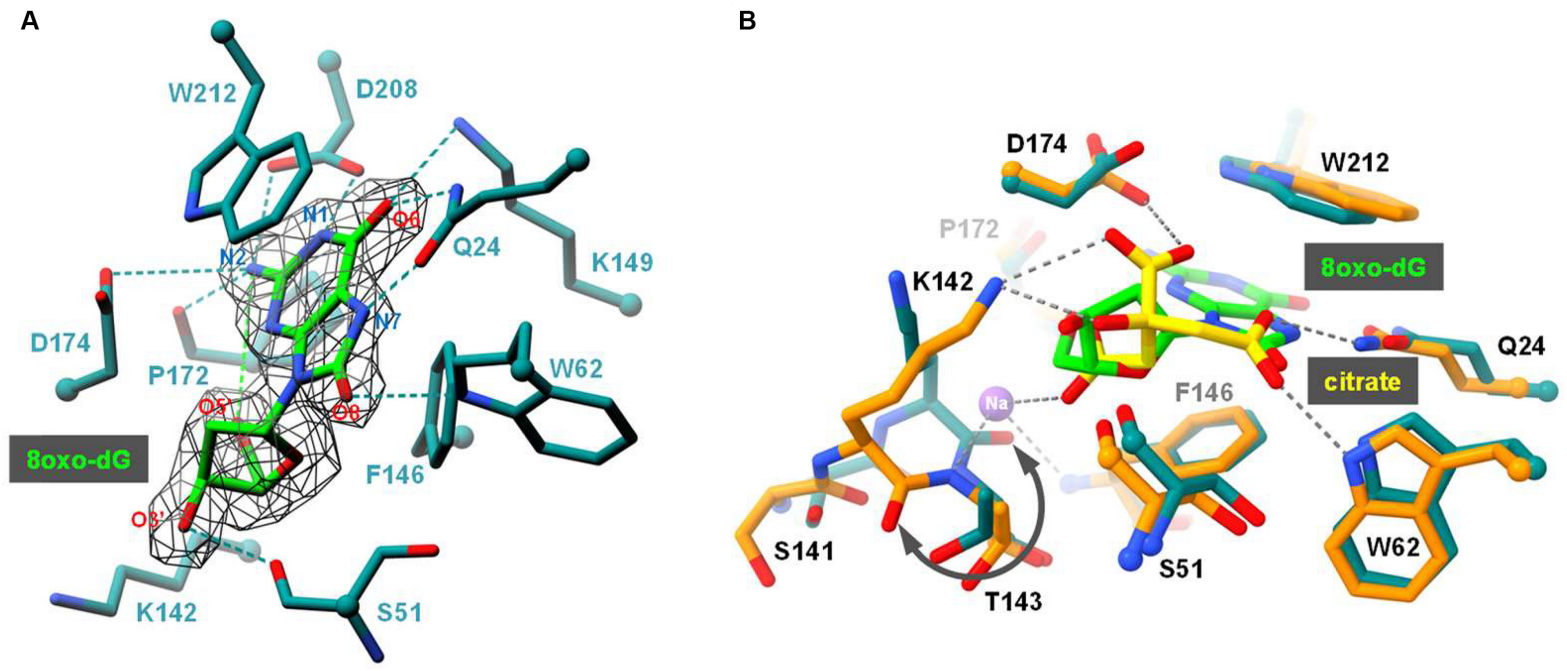
Overview of the Pab-AGOG binding site. (**A**) The 8oxo-dG is shown in *green*. Pab-AGOG interacting residues and the catalytic lysine (K142) are represented as *blue* sticks (but Cα as balls). Electron density mFo-DFc composite omit map is contoured at 5σ. Hydrogen bonds are shown with dashed lines. (**B**) Superposition of apo (*orange* and *yellow*) and 8-oxodG bound (*blue* and *green*) Pab-AGOG.

Currently, the only two AGOG protein structures found in the PDB are those of Pfu- (*Pyrococcus furiosus*) and Pae-AGOG (*Pyrobaculum aerophilum*). A BLASTp search (43) showed that the Pab-AGOG (*Pyrococcus abyssi*) and Tga-AGOG (*Thermococcus gammatolerans*) sequences are more closely related to that of Pfu- than that of Pae-AGOG (Figure 2). To shed more light on structure and function of these AGOGs, the X-ray 3D structures of Pab-AGOG and Tga-AGOG were solved by molecular replacement using PDBid 1XG7 as a search model (Table 1, Pab-AGOG and Tga-AGOG) (20). Like other AGOGs and OGG2, but unlike OGG1, which contains some β-sheets, Pab-AGOG folds into an all-α protein with an *N/C* (N and C termini) domain and a *HhH* (helix-hairpin-helix) domain (Figure 3A). The *N/C* domain is composed of six α-helices (α1, α2, α11, α12, α13, α14) and the two chain ends. The *HhH* domain consists of eight α-helices in a row (α3–α10) and contains the *HhH* motif found in many DNA-binding proteins (44). As expected from the sequence alignment, the 3D structures of Pfu-AGOG and Pab-AGOG are closely related with a rmsd value of 1.2 Å over all Cα atoms (Figure 3B-C). The comparison of Pae-AGOG and Pab-AGOG 3D models brings out conformational differences in the *N/C* domain (rmsd value of 3.5 Å) resulting from an insertion of twelve amino acids in Pae-AGOG between α11 and α12 helices and in the *HhH* domain (rmsd value of 2.2 Å, Figure 3C) due to two residue deletions in the Pae-AGOG primary sequence (Figure 2B). None of these additional residues were predicted to interact with DNA or to be engaged in the catalytic mechanism of the enzyme (20). The residues involved in GO-moiety recognition are conserved among the AGOG proteins studied so far (Figure 2A), and we confirmed by soaking crystals in crystallization buffer containing 8-oxoguanosine (8-oxodG, Table 1, Pab-AGOG + 8-oxodG) that the Pab-AGOG binding pocket has the same conformation as the one previously observed for Pae-AGOG (Figure 4A). The damaged base is sandwiched between two aromatic residues (F146 and W212) and makes H-bonds with six Pab-AGOG residues (Q24, W62, K149, P172, D174, D208). The sugar moiety of 8-oxodG interacts through its O3’ with the carbonyl group of S51 while the O5’ establishes an intra-residual H-bond with the N2 atom of the base. Unlike Pae-AGOG, Pab-AGOG shows a change of backbone conformation at the catalytic K142 residue upon GO binding. The torsional angle Ψ of K142 has a value of 128.8° in the apo structure and of –42.9° when complexed to GO, leading to a substantial conformational change of the *HhH* hairpin. This conformational change could be induced and/or stabilized by the presence in the protein active site of sodium citrate coming from the crystallization buffer (Figure 4B). Indeed, citrate interacts directly with residues Q24, W62, K142 and D174 and a sodium ion which is close to the position of the K142 carbonyl group in the Pab-AGOG/8-oxodG structure. Another difference between Pab-AGOG and Pae-AGOG concerns a major conformational change of the flexible segment L61-K64 (short loop between α4 and α5 helices) observed in Pae-AGOG upon 8-oxodG binding. An equivalent difference between apo and bound states is not seen for Pab-AGOG, likely due to the presence of citrate in the active site of the apo Pab-AGOG and its stabilising effect on the flexible segment through an H-bond to W62.

Sequence alignment of AGOG proteins in Figure 2 shows that the main difference between Pab- and Tga-AGOG occurs at one end of the molecules. Tga-AGOG has an extension of 11 or 19 residues at the *N*-terminus compared to Pae-AGOG or Pab-AGOG, respectively. This non-structured part of the protein was cleaved between residues R8-I9 by trypsin and the first residue that could be located without ambiguity in the electron density map was Y11 (Trypsin was used to remove the His-Tag because the internal proteolytic site between the Tag and the N-terminus of Tga-AGOG is thrombin resistant). Another part of Tga-AGOG appears to be more flexible than in Pab- and Pae-AGOG. In the two molecules present in the asymmetric unit, residues between L236 and G240 were not clearly visible and were not assigned due to high mobility. In fact, the mean *b*-factor for the *N/C* domain (43 Å^2^) is almost twice as high as that observed for the *HhH* domain (23 Å^2^). This observation could be related to the absence of a disulfide bridge between the two cysteine residues present in Tga-AGOG (C32 and C250, hypothetical S/S in Supplementary Figure S5A), despite the fact that they are close to each other in the 3D structure (3.54 Å). On the other hand, Pae-AGOG has three disulfide bridges, one stabilizing the *N/C* domain (C181-C251) and two located in the *HhH* domain (C36-C155, C85-C113) (Supplementary Figure S5A). Interestingly, Pab-AGOG lacks cysteine residues and therefore does not require disulfide bridges to stabilize a functional structure at high temperature. Despite differences in the number of disulphide bridges, Tga-AGOG adopts the same conformation as Pab- or Pae-AGOG with minor differences located mainly in the *N/C* domain. In an attempt to assess the role of the potential disulfide bridge in Tga-AGOG and the three disulfide bridges observed in the structure of Pae-AGOG, we compared the GO-DNA glycosylase activity of the three AGOGs after their pre-incubation at 20°C or 80°C in the presence or absence of the thioreducer dithiothreitol (DTT) (Supplementary Figure S5B). After a pre-treatment at 80°C, only the reduced Pae-AGOG form shows a significant loss of activity indicating that Pae-AGOG absolutely needs its disulfide bridges to be functional at high temperature. Among the three bridges of Pae-AGOG, C181-C152 (SS1 in Supplementary Figure S5B) appears the most critical for stabilizing the *N/C* domain in particular in the vicinity of the insertion of twelve amino acids between α11 and α12 helices of the enzyme. Under the same conditions, the oxidized and reduced forms of Tga-AGOG are indistinguishable in terms of their activity (at least up to 80°C), indicating that this enzyme, like Pab-AGOG, does not rely on disulphide bridges and instead achieves heat resistance by means that are insensitive to a reducing agent. Differential reliance on disulphide bridges might reflect different environmental conditions (in terms of the redox potential or sulphur availability, (45)) in which these three archaeal proteins have evolved.

### Structure of the borohydride-trapped complex of Pab-AGOG bound to single-stranded DNA

**Figure 5.**
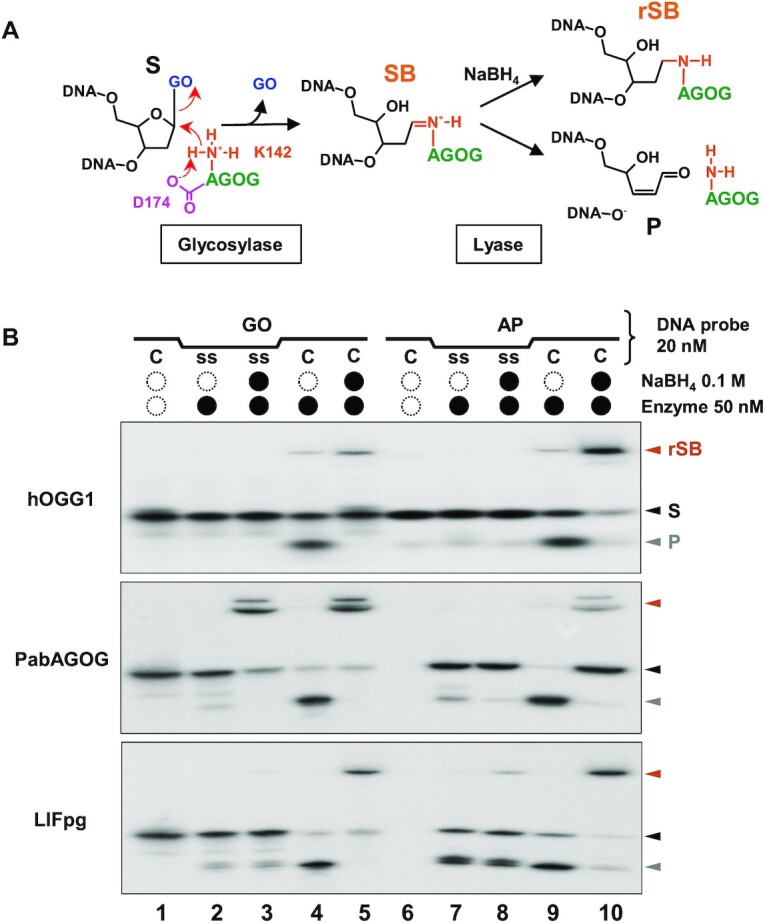
Trapping of the imino Pab-AGOG-DNA covalent intermediate. (**A**) Schematic representation of the GO-DNA glycosylase/AP lyase catalytic mechanism. K142 and D174 are the catalytic residues of Pab-AGOG. (**B**) Comparative trapping assays with Pab-AGOG, hOGG1 and LlFpg. As indicated, 20 nM of 5’[^32^P]-labeled GO- or AP site-containing single (ss-GO or ss-AP) or double-stranded (GO:C or AP:C) 24-mer DNA fragment was incubated for 15 min at 37°C alone (lanes 1 and 6), with 50 nM of indicated enzyme only (lanes 2, 4, 7 and 9) and 0.1 M NaBH_4_ (lanes 3, 5, 8, 10). Reactions were then analyzed by urea–PAGE as described in Materials and Methods. Representative autoradiographs are presented. The stable reduced Schiff base (rSB) is indicated by an *orange* arrow and, S and P are for DNA substrate and cleaved end-product, respectively.

As established previously, bifunctional DNA glycosylases from the HhH-GPD structural super family including AGOGs use an active lysine as a catalytic residue to perform both the glycosylase and AP lyase catalytic steps. This involved a nucleophilic attack of the C1’ of the damaged nucleotide by the activated ϵ-amino group of the catalytic lysine (Figure 5A) resulting in the intermediate formation of an abasic (AP) site covalently bound to the enzyme (a transient imino enzyme-DNA intermediate or a protonated Schiff base, SB). Due to the presence of a labile proton at the C2’ of the sugar and of a good leaving group at position beta of C1’, the lyase process consists in the cleavage of the phosphodiester bond at the 3’ side of the AP site by a β-elimination mechanism. SB can be easily trapped by its irreversible stabilization with a strong reducing agent such as sodium borohydride (NaBH_4_). The catalytic lysine of AGOG has been identified previously in the C-terminal part of the hairpin of the *HhH* motif as K140 in Pae-AGOG (18). The sequence alignment based on 3D structures unambiguously shows that the catalytic lysine is strictly conserved in AGOGs and corresponds to K142 and K162 of Pab-AGOG and Tga-AGOG, respectively (Figure 2). As expected but not shown previously for other AGOGs, the imino enzyme-DNA intermediated (Schiff base, SB) or more exactly its reduced form (rSB) can be easily observed with Pab-AGOG incubated with GO-containing single- or double-stranded DNA in the presence of NaBH_4_ (lanes 3 and 5, Figure 5B). Under the same condition, rSB is not observed with hOGG1 and GO-containing single-stranded DNA which is completely consistent with the fact that this enzyme is efficient only on double-stranded DNA (Figure 1). For Fpg, rSB is barely detected, probably because Fpg is less efficient than AGOG at excising GO from single-stranded DNA. In similar experiments using AP site-containing single-stranded DNA as a substrate (lanes 6–8, Figure 5), we observed rSB only with Fpg, in agreement with the observation that AGOGs and hOGG1 are inefficient at cleaving the AP site in single-stranded DNA (lanes 11–15, Figure 1).

**Figure 6.**
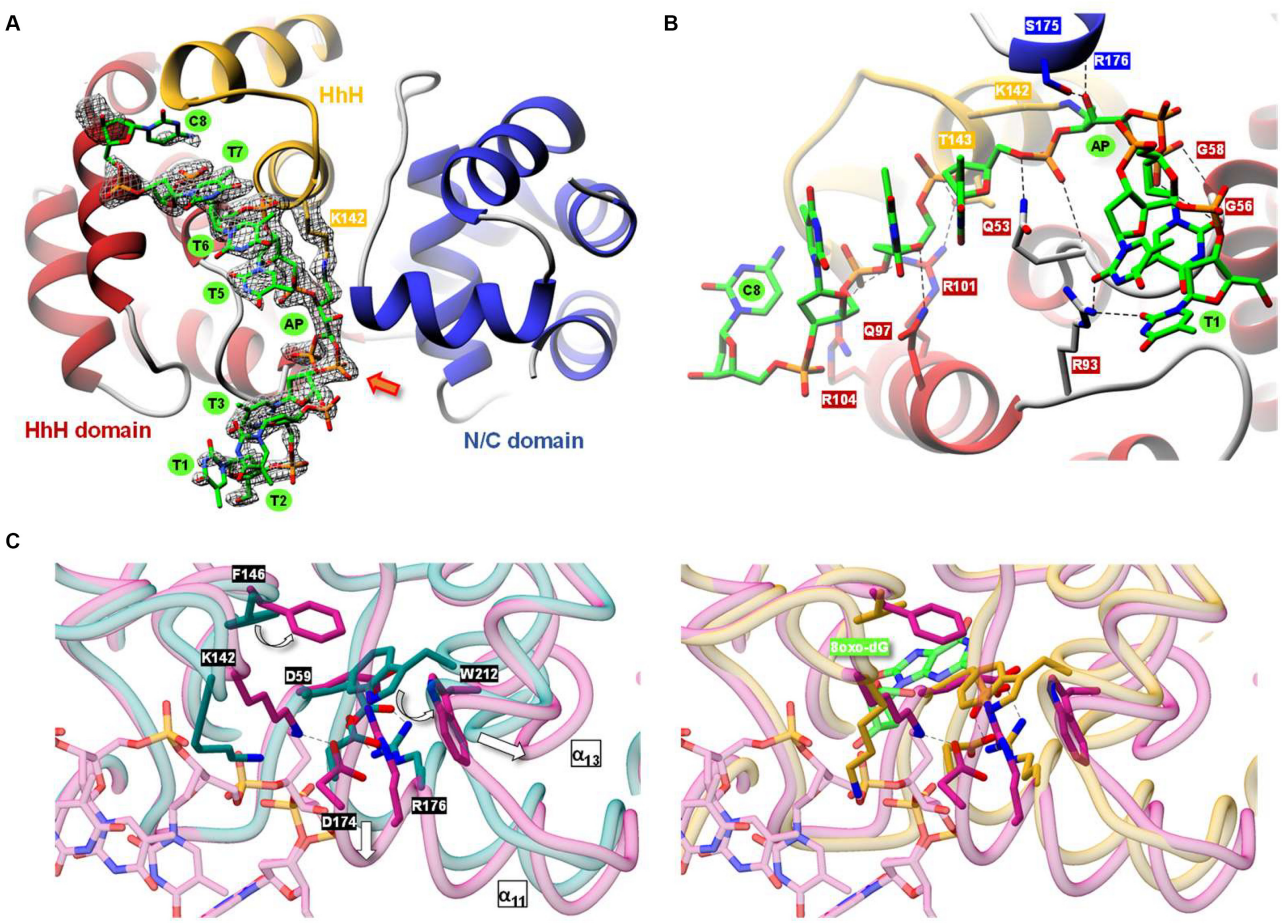
3D structure of Pab-AGOG trapped to single-stranded DNA. (**A**) Overwiew of the complex. mFo – DFc simulated annealing omit map contoured at 3σ (DNA and K142 residue omitted) is shown as black mesh. The phosphate group with two conformations is denoted by an arrow. (**B**) Details of the direct interactions between Pab-AGOG and single-stranded DNA. H-bonds are represented as *black* dash lines. (**C**) Superposition of the single-stranded DNA trapped on Pab-AGOG (*violet*) with apo Pab-AGOG (*dark cyan*) or Pab-AGOG + 8oxodG (*yellow*/*green*).

To get structural insight into AGOG bound to single-stranded DNA, we crystallized rSB using Pab-AGOG and GO-containing single-stranded DNA. rSB obtained between Pab-AGOG and a 13-nucleotide single-stranded DNA containing a GO at position 7 (Supplementary Figure S1) in the presence of NaBH_4_ was purified to homogeneity as described in Materials and Methods. Following numerous unsuccessful crystallization attempts, we decided to shorten the single-stranded DNA used (Supplementary Figures S1 and S6). Best diffracting crystals were obtained with a 9-mer single-stranded DNA, allowing us to solve the structure of Pab-AGOG borohydride-trapped-DNA complex (Table 1, Pab-AGOG_ssDNA). Four complexes were present in the crystal asymmetric unit, each showing a slightly different conformation for the DNA molecule (Supplementary Figure S7). Figure 6A shows the 3D structure of one of the complexes, which will be used as the basis for the description of protein-DNA interactions. As expected, the covalent bond between the ϵ-amino group of K142 and the C1’ of the ring-open form of the AP site is clearly seen from the electron density confirming K142 as the catalytic nucleophile of Pab-AGOG (Figure 6A, and Figure 5A). Few nucleotides are contacted by the enzyme: 2 and 3 nucleotides at the 5’ and 3’ side of the AP site, respectively. The single-stranded DNA is bound in the groove between the two protein domains and the *HhH* motif. Pab-AGOG interacts *via* its R93 residue with the T1 and T2 bases at the 5’ DNA extremity and *via* the Q53, G56, G58, Q97, R101, R104, T143 residues with the DNA phosphodiester backbone or deoxyribose of the residues PED4 (AP site) to T7 (Figure 6B, Supplementary Figure S7). At the trapped AP site, the phosphate group fluctuates between two conformations and a large kink of about 90° is observed for the DNA backbone. In the structure, the AP site is stabilized by Oγ of S175 and the amide of the strictly conserved R176, which form hydrogen bonds with the O4’ hydroxyl group. Compared to the apo or 8-oxodG-bound structures, the protein sidechains of residues F146 and W212 show severe rearrangement concomitant with the displacement of the helices α11 and α13 of the *N/C* domain. The tilting of the W212 indole-moiety is stabilized by the R176 residue which adopts a new conformation after the breakdown of the salt bridge that it formed with D59 in the apo state. Pae-AGOG harboring the single substitution D172Q or D172N (D174 in Pab-AGOG) in the *GPD* motif has no detectable activity (19). Interestingly, the movement of the α11 helix allows the catalytic residue D174 of Pab-AGOG to be close to the nucleophile K142 which is responsible for the covalent Schiff base intermediate (Figure 6C). Based on the present 3D structure, we can propose that D174 could be responsible for the deprotonation of the ϵ-amino group of K142 and, therefore, directly involved in the activation of K142 for catalysis (Figure 5A).

### AGOG selectivity for the base opposite the damage

**Figure 7.**
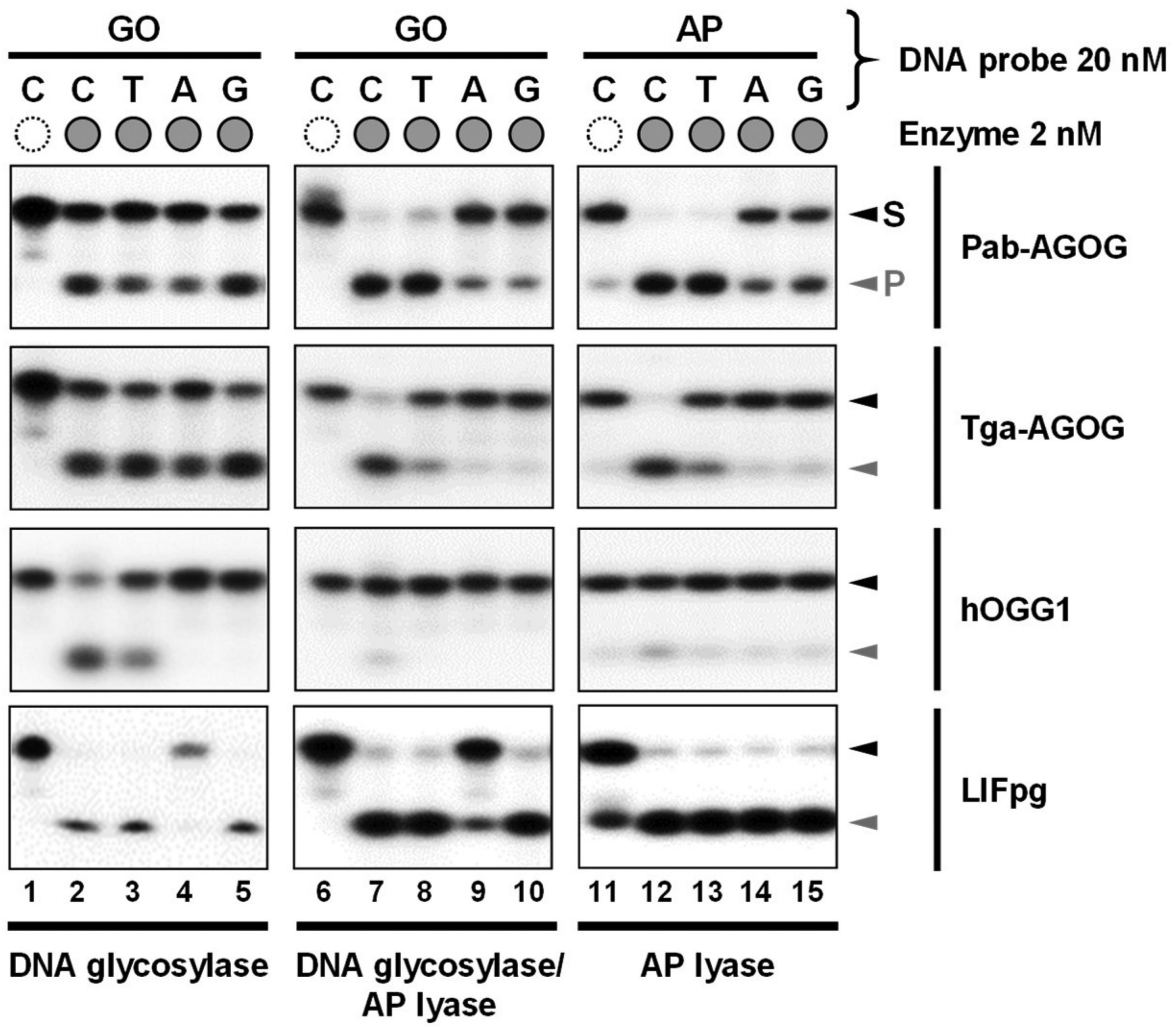
Comparative analysis of the effect of the base opposite the damage on the activities of several GO-DNA glycosylases under MTO conditions. As indicated, 20 nM of radiolabeled 24-mer DNA duplex containing GO or AP site opposite C, T, A or G was incubated at 37°C alone (empty dashed circles, GO:C and AP:C, lanes 1 and 6, and 11, respectively) or with 2 nM enzyme (full grey circles; lanes 2–5, 7–10 and 12–15 for DNA glycosylase, DNA glycosylase/AP lyase and AP lyase activity, respectively). Incubation times were: 2 min for DNA glycosylase of LlFpg, 5 min for DNA glycosylase of Pab- and Tga-AGOG and for DNA glycosylase/AP lyase and AP lyase of LlFpg, 20 min for DNA glycosylase/AP lyase and AP lyase of Pab-AGOG, 60 min for all activities of hOGG1 and for DNA glycosylase/AP lyase and AP lyase of Tga-AGOG. Reaction mixtures were then analyzed by urea–PAGE as described inMaterials & Methods. Representative autoradiographs are shown.

As both GO and AP sites are mutagenic lesions, it is usual to assess the effect of the nature of the base opposite the damage in double-stranded DNA on the GO-DNA glycosylase activity and DNA binding (7,46–48). We investigated first the DNA glycosylase, DNA glycosylase/AP lyase and AP lyase activities in single- and multi-turnover conditions, STO and MTO, respectively (Figure 7, Supplementary Figure S8 and Table 2). In these experiments, we chose to keep the protein/DNA ratio constant (10 and 0.1 for the STO and MTO experiments, respectively) and limit the incubation times in order to be able to better probe potentially subtle effects of the base opposite the damage.

**Table 2.** Single turnover rate constant of Pab-AGOG and Tga-AGOG (*k*_obs_) for GO- and AP site-containing single or double stranded DNA and its modulation by the base opposite the damage

		*k*_obs_ (min^−1^) at 37°C		
Enzyme	Subtrate	DNA glycosylase	DNA glycosylase/ AP lyase	AP lyase
**Pab-AGOG**	dsGO/C	>15	0.63 ± 0.02	NA
	dsGO/A	>15	0.37 ± 0.01	NA
	dsGO/G	>15	0.35 ± 0.01	NA
	dsGO/T	>15	0.39 ± 0.02	NA
	ssGO	6.7 ± 0.4	NM	NA
	dsAP/C	NA	NA	1.51 ± 0.08
	dsAP/A	NA	NA	1.02 ± 0.03
	dsAP/G	NA	NA	0.45 ± 0.02
	dsAP/T	NA	NA	7.95 ± 0.30
	ssAP	NA	NA	NM
**Tga-AGOG**	dsGO/C	>20	0.221 ± 0.011	NA
	dsGO/A	>20	0.053 ± 0.001	NA
	dsGO/G	>20	0.050 ± 0.001	NA
	dsGO/T	>20	0.217 ± 0.009	NA
	ssGO	>20	NM	NA
	dsAP/C	NA	NA	0.332 ± 0.010
	dsAP/A	NA	NA	0.051 ± 0.001
	dsAP/G	NA	NA	0.051 ± 0.001
	dsAP/T	NA	NA	0.344 ± 0.001
	ssAP	NA	NA	NM

NA = not applicable; NM = not measurable because the reaction rate is too low to be measured under the chosen conditions. The *k*_obs_ values were extracted from three independent kinetic experiments (Supplementary Figure S3).

Unlike for hOGG1, the DNA glycosylase activity of Pab-AGOG and Tga-AGOG appears to be only slightly affected by the base opposite GO in STO condition (lanes 2–5; Supplementary Figure S8) as it has been already observed for OGG2 (51). These results are in agreement with previous studies with Pae-AGOG and Tko-AGOG (19,23). For the concerted DNA glycosylase/AP lyase activity, similar results were observed for Pab-AGOG (Supplementary Figure S8, lanes 6 to 10), while Tga-AGOG displays a marked preference for a DNA substrate containing GO opposite a pyrimidine. The *k*_obs_ values of the DNA glycosylase/AP lyase obtained for Tga-AGOG and Pab-AGOG are in the same range of magnitude as those previously determined for Tka-AGOG (23). In STO, the AP lyase activity of Pab-AGOG is partially inhibited by a purine opposite the AP site and Tga-AGOG is strongly inhibited in the same circumstances, similarly to hOGG1 (Supplementary Figure S8, lanes 11–15) (16,49,50). The high sensitivity of the AP lyase activity and, to a lesser extent, of the concerted DNA glycosylase/AP lyase activity of AGOGs to the base opposite the damage may correlate with the inability of AGOGs to cleave the AP site in single-stranded DNA (Figure 1). Under the same conditions, only LlFpg used as control appears to be indifferent to the base opposite the AP site, but the rate of the reaction is too high to really appreciate this. In STO conditions, it is not possible to assess the effect of the base opposite the damage because the GO excision rate (DNA glycosylase) by Pab-AGOG and Tga-AGOG is too high to determine the rate constant *k*_obs_ reliably (Table 2). Pab-AGOG and Tga-AGOG appear much faster than what was previously observed for Tko-AGOG and Pae-AGOG (19,23), which might be due to our use of 0.1% BSA in assay reactions, which is known to stabilize enzymes at very low concentrations. If there is an effect on glycosylase activity, it is probably smaller than for the AP lyase activity and could be partly correlated with the fact that AGOGs are able to efficiently excise GO from ssDNA (*k*_obs_ = 6.7 ± 0.4 and >20 min^−1^ for Pab- and Tga-AGOG, respectively). A pyrimidine (to a lesser extent a T for Tga-AGOG) opposite the lesion is the better substrate for AGOGs, as already observed previously for Tga-AGOG (21). As expected, the *k*_obs_ values of Tga-AGOG’s DNA Glycosylase/AP lyase and AP lyase activities are considerably lower (at least 4 and 6 times, respectively) for substrates containing a purine opposite the lesion compared to those with a pyrimidine in that position (Table 2). For Pab-AGOG, the discrimination of the base opposite the damage is much less marked than for Tga-AGOG as already observed for Pae-AGOG and AGOG from *Thermococcus kodakarensis* (Tko-AGOG) in STO conditions (19,23). In this respect, AGOGs differ strongly from OGG1 enzymes whose best substrate is by far the one with a C opposite the damage (Supplementary Figure S8) (51,52).

In MTO conditions, the DNA glycosylase activities of Pab-AGOG and Tga-AGOG are slightly affected by A or T and A opposite GO, respectively (Figure 7: lanes 1–5). As expected, hOGG1 only tolerates a C (and to a lesser extent a T) opposite GO, whereas LlFpg is only slightly affected by an A opposite GO, similarly to its *E. coli* Fpg homolog (49,50,53). The DNA glycosylase/AP lyase and AP lyase activities of AGOGs are strongly inhibited by a purine opposite the lesion and to a lesser extent by a T for Tga-AGOG. In conclusion, the statement in the literature that the activity of AGOGs is insensitive to the base opposite the damage is rather true for the DNA glycosylase activity but not for the Glycosylase/AP lyase and AP lyase activities. Among GO-DNA glycosylases studied so far, AGOGs present an intermediate selectivity for the base opposite the damage (weakly manifested for GO but more strongly for the AP site) positioning it from this point of view between OGG1 removing/cleaving GO/AP site only paired with C and OGG2 that works on these lesions regardless of the nature of the opposite the damage (24,52,54,55).

In order to better clarify whether this effect on catalysis may be due to differences in the ability of AGOGs to recognize DNA substrates, we performed EMSA to determine the apparent dissociation constants (*K*_Dapp_) of Pab-AGOG and Pae-AGOG for GO or tetrahydrofuran (THF, a stable AP site analogue) opposite C or A (Supplementary Figure S9, Table 3). Although most of the ligands tested form stable complexes with the two enzymes, Pab-AGOG has a much higher affinity for DNA than Pae-AGOG. Thus, Pab-AGOG (K241Q) recognizes GO opposite C 650 times better than Pae-AGOG (WT). Similarly, Pab-AGOG (WT/K241Q) recognizes THF opposite C 200 times better than Pae-AGOG (WT). However, large differences in the affinity of the two AGOGs for double-stranded DNA depending on the base opposite the damage are observed. As shown previously for other AGOGs, the catalytically defective mutant K142Q retains similarly high affinity for lesion-containing DNA to that of the WT enzyme. DNA containing THF or GO opposite C are ligands of higher affinity than those having lesions opposite A (Table 3). For example, Pab-AGOG (WT) recognizes dsTHF:C with an affinity 88 times greater than that of dsTHF:A. Pab-AGOG (K142Q) is 50 times more affine for dsGO:C than for dsGO:A. Thus, the base opposite THF can affect the affinity of Pab-AGOG much more than the base opposite GO does, as already suggested by enzyme catalytic assays, where the AP lyase activity of Pab-AGOG is more sensitive than its GO-DNA glycosylase activity to the base opposite the damage (Figure 7). Interestingly, Pab-AGOG (the catalytically defective K142Q mutant) has 9 times higher affinity for ssGO than for dsGO:A and only 4.5 times higher for dsGO:C than for ssGO, establishing ssGO as a high affinity ligand for Pab-AGOG as compared to dsGO:A. In the range of protein concentration used, no binding is observed with ssTHF, which is consistent with the inability of the protein to cleave the AP site in single-stranded DNA (Figure 1).

**Table 3.** Apparent dissociation constants of wild type and mutants Pab-AGOG and Pae-AGOG for GO or THF (AP site analog)-containing single- or double-stranded DNA probes

	*K*_Dapp_ (nM)				
	Pab-AGOG			Pae-AGOG	
DNA probe	WT	K142Q	R93A	WT	R60A
**dsGO/C**	NA	0.097 ± 0.065	51.0 ± 1.6	63.0 ± 1.6	3450 ± 616
**dsGO/A**	NA	4.85 ± 0.59	0.625 ± 0.018	151.0 ± 8.8	NB
**ssGO**	NA	0.53 ± 0.025	LB	LB	LB
**dsTHF/C**	0.163 ± 0.065	0.095 ± 0.005	78.7 ± 2.51	19.9 ± 0.6	1150 ± 40
**dsTHF/A**	14.3 ± 1.5	1250 ± 331	LB	NB	NB
**ssTHF**	NB	NB	NB	LB	NB

NA = not applicable (under the conditions used GO-containing duplexes are partially processed by Pab-AGOG-WT, see Supplementary Figure S12); NB = no binding; LB = low binding (formation of several unstable complexes, *K*_Dapp_ > 10 μM). The *K*_Dapp_ values were extracted from three independent EMSA titration experiments (Supplementary Figure S4 and S9).

**Figure 8.**
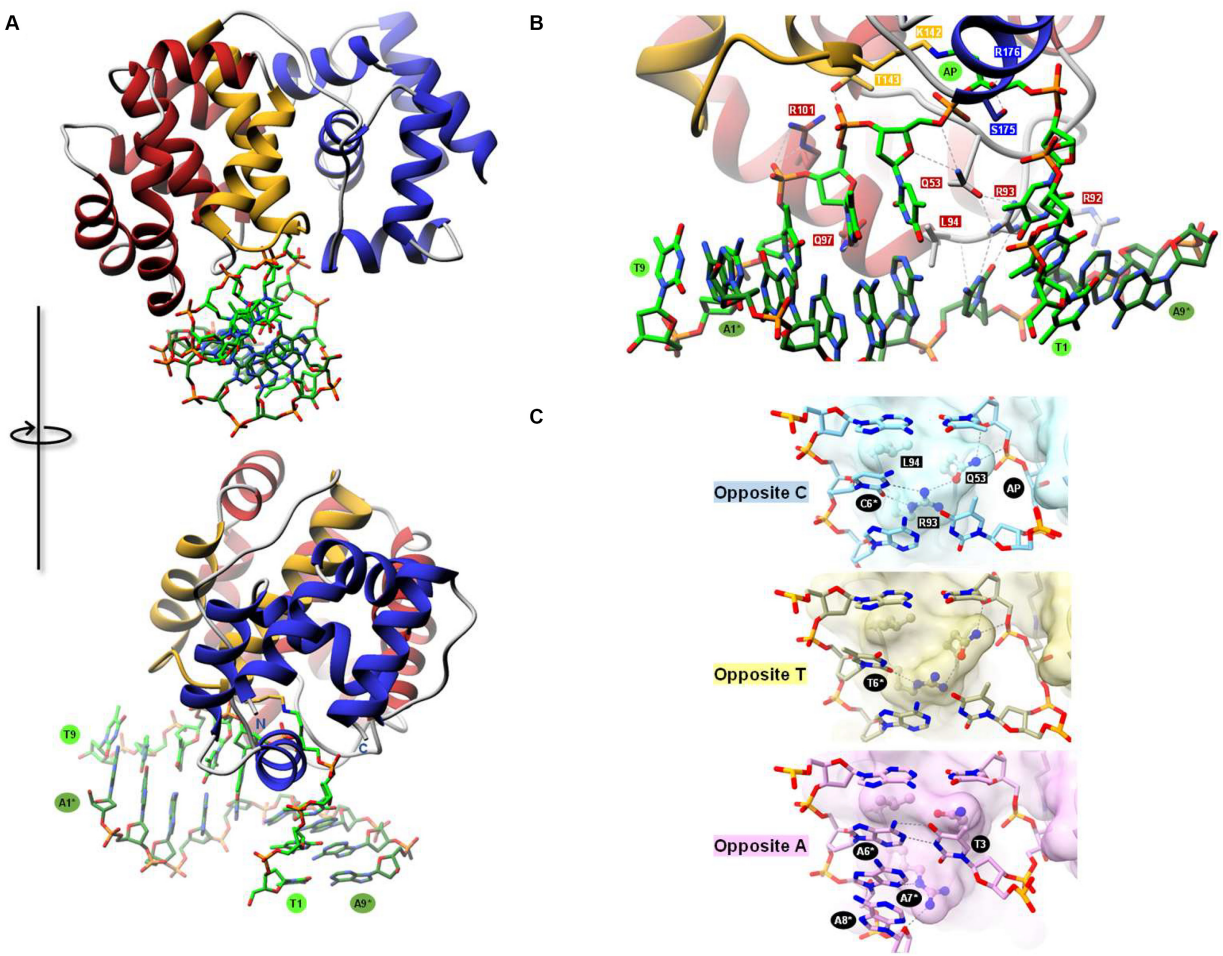
3D structure of covalently trapped Pab-AGOG-AP-dsDNA complexes. (**A**) Overview of the Pab-AGOG/AP dsDNA-C complex. (**B**) Details of the direct interactions between Pab-AGOG and AP dsDNA-C. (**C**) Opposite base recognition.

With the aim of better deciphering the interaction mode of AGOG with double-stranded DNA, the 3D structure of a borohydride-trapped Pab-AGOG on a double-stranded DNA with a C opposite the lesion was solved to 1.70 Å resolution (Table 1, Pab-AGOG_dsDNA-C). The overall structure of the complex is shown in Figure 8A. Pab-AGOG intercalates three residues (Q53, R93 and L94) inside the DNA minor groove inducing a sharp bending (∼65°) of the duplex centred on the lesion site. Several protein residues establish DNA backbone interactions with phosphates bordering the trapped AP site and with the two other successive phosphates at the 3’-side of the AP site and with the two phosphates at the 5’-side of the orphan C on the opposite strand (Supplementary Figure S10). The AP site-containing strand shows minor changes of the interaction network compared to that observed in the structure of Pab-AGOG trapped with the single-stranded DNA (Supplementary Figure S10A,B). S175, R176 and Q53 establish H-bonds with the AP lesion, T143 and R101 make hydrophilic contacts with the phosphate group of T5 and T6, whereas Q97 contacts the O4’ of the T6 deoxyribose. Only two residues of Pab-AGOG interact with the DNA opposite strand. R92 (substituted by a lysine in Pae-AGOG) is H-bounded to phosphate groups of A7* and A8* at the 3’ side of the damage opposite strand and L94 wedges its sidechain into the space between the orphan C (C6*) and the adenine A5* on its 5’ side and through its mainchain amide group makes an H-bond with the O4’ of C6*. Thereby, L94 unstacks A5* and C6* and induces a local sharp DNA kink, making it easier to access the Watson–Crick edge of the base through the minor groove. The Pab-AGOG-induced DNA curvature is stabilized by the intercalation triad Q53-R93-L94, which fills in the large space resulting from the GO excision, thus preventing the DNA structure from collapsing at the damage site. In hOGG1 and OGG2, an equivalent role is played by an aromatic residue, Y203 and F85, respectively (24,54). The R93 sidechain establishes two H-bonds with the acceptor N3 and O2 atoms of the estranged C and stacks on top of its 3’-neighbour. The R93 sidechain position is also ideal to interact with Q53, which makes H-bonds with R93 and a phosphate (T5) of the lesion containing strand. This interaction network establishes a strong physical link between the estranged C and the damage-containing strand, preserving the orphan pyrimidine inside the DNA double helix (Figure 8B). Thus, two Pab-AGOG residues (R92, L94) are mobilized to bind (*via* 3 H-bonds) and maintain the orphan C nucleotide in an intrahelical *anti*-conformation while OGG1 and OGG2 use three residues (N149, R154, R204, involving 5 H-bonds that specify only a C opposite the damage) and only one residue (R84, equivalent to R204 and R93 of hOGG1 and Pab-AGOG, respectively, involving 2 H-bonds), respectively (24,54). Surprisingly, the side and main chains of the triad residues are already positioned correctly for DNA binding in the apo Pab-AGOG. This is analogous to the case of OGG2, whose R84 also anticipates its DNA-bound conformation already in the DNA-free state (53). On the other hand, the situation is different for OGG1, where the equivalent residues N149 and R204 are finely reoriented toward the C opposite the lesion upon DNA binding (24,56). From this point of view, previous structural and functional studies provide an unambiguous explanation for the very narrow selectivity of hOGG1 for a C opposite the lesion and the broad tolerance of OGG2 for any base opposite the lesion. For Pab-AGOG, the present structural and biochemical data suggest an intermediate situation between OGG1 and OGG2, which remains to be more documented.

To shed light on the opposite base selectivity at the atomic level, we also solved the 3D structures of the trapped Pab-AGOG-dsDNA harbouring either a T (Pab-AGOG_dsDNA-T) or an A (Pab-AGOG_dsDNA-A) opposite AP site (Table 1). Crystallization trials to obtain the structure of a complex with an opposite G were unsuccessful. The details of the protein/DNA interactions at the lesion site are shown in Figure 8C. In structure with a T opposite the damage, residues of the intercalated triad show the same position as the one in the structure with an opposite C, but R93 is only able to make one H-bond with the base through its Nϵ atom. This loss of interaction has at most a minor effect on the interaction network architecture (opposite base-R93-Q53-damaged strand), which strongly constrains the relative orientation of T opposite the damaged strand. Concerning the structure with an A opposite the damage, the mode of interaction is less straightforward and clearly independent from the R93 interactions. Pab-AGOG appears to have no difficulty accommodating the steric hindrance of adenine in an intrahelical *anti*-conformation, but significant differences from the two structures described above for both protein and DNA conformation at the lesion site are observed. Indeed, the opposite A forms a Watson–Crick base pair with T3 of the lesion-containing strand leading to the repositioning of the Q53 and R93 sidechains. This binding-induced structural readjustment results in the loss of the interaction network linking the opposite base to the damaged strand that was observed in the structures with C or T opposite the damage. R93 is no longer able to establish contacts with the base opposite the lesion, nor with Q53, and its sidechain reorients to interact with A7* and A8* of the opposite strand, leaving space for the adenine opposite the lesion. In this structure, one should note that T1 and T2 base pair with A8* and A7* instead of A9* and A8*, respectively, as was the case for structures with C or A opposite the damage. This structural remodelling of the lesion recognition complex could be associated with the significant loss of affinity of Pab-AGOG for the AP site opposite A observed above (Table 3). Overall, these structural models with C, T or A opposite the damage do not offer a simple explanation why the GO-DNA glycosylase activity of Pab-AGOG is less sensitive to the nature of the base opposite the damage. They do, however, indicate a high plasticity of the Pab-AGOG DNA binding site, which can accommodate a purine opposite the damage in an intrahelical conformation.

It should be noted that the intercalation triad residues (Q53-R93-L94) are often—but not strictly—conserved in the AGOG family. Sequence alignment based on 3D structure suggests that Q53, R93 and L94 of Pab-AGOG (also found in Tga- and Pfu-AGOGs) are substituted by R60, I100 and G101 in Pae-AGOG, respectively (Figure 2). For Pae-AGOG the recognition of the estranged C should be different from that of Pab-AGOG and might involve the residue R60 (Supplementary Figure S11). R93 of Pab-AGOG and R60 of Pae-AGOG might be expected to play a similar key role in substrate recognition. To evaluate this hypothesis, we mutated these two residues to alanine and compared the affinity of these resultant Pab- and Pae-AGOG variants for different DNA duplexes with those of the corresponding wild-type proteins (Table 3). Pab-AGOG-R93A and Pae-AGOG-R60A variants are both affected in their GO-DNA glycosylase activity confirming the key role of the mutated residues in the enzyme catalytic and/or binding (Supplementary Figure S12). This strong decrease in activity is associated with a severely diminished ability to recognize double-stranded DNA containing a C opposite GO or AP site, by a factor of more than 500 and 50 for Pab-AGOG and Pae-AGOG, respectively. As expected from the structural model of Pab-AGOG bound to a double-stranded DNA, R93 (and R60 of Pae-AGOG) are essential for stabilizing the lesion recognition complex of Pab-AGOG and Pae-AGOG, respectively. Concerning the selectivity of the variants R93A and R60A for the base opposite the damage, no stable complex could be demonstrated by EMSA when the damage is positioned opposite a purine. We can conclude from these experiments that R93 and R60 probably contribute, at least weakly, to AGOGs’ ability to stabilize the lesion recognition complex whatever the base opposite the damage and, perhaps, especially if that base is a purine. However, the structure of the lesion recognition complex with Pae-AGOG must be different to that of Pab-AGOG because, in the free enzyme, R60 occupies a space in the binding site that is very different from that taken by R93 in Pab-AGOG, which would likely lead to the reduction of space left vacant by the extrusion/excision of the damaged base. This hypothesis could explain why Pae-AGOG is more sensitive than Pab-AGOG to the nature of the base opposite the damage, even if the Pab-AGOG selectivity is less marked for a purine opposite GO.

### Structural determinants of Pab-AGOG bound to GO-containing DNA and substrate specificity

**Figure 9.**
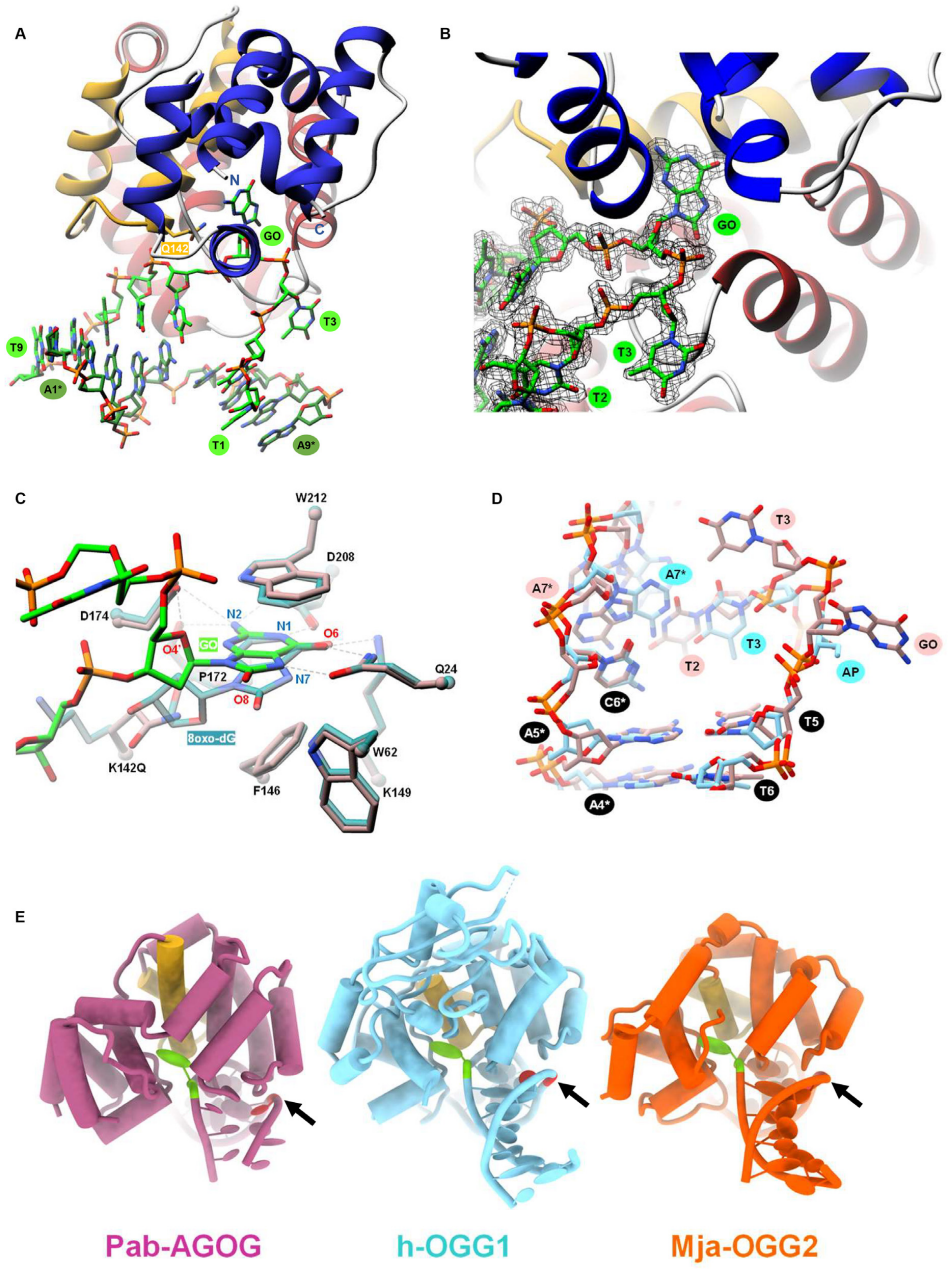
3D structure of K142Q-Pab-AGOG complexed with GO-containing double-stranded DNA. (**A**) Overview of the Pab-AGOG/dsDNA-GO:C complex. (**B**) Zoom of the double base flipping (GO and T3) in the K142Q-Pab-AGOG complex. Around the DNA, the 2Fo – Fc electron density map contoured at 1σ is shown as *black* mesh. (**C**) Close-up view of the binding pocket of the Pab-AGOG/dsDNA-GO:C complex (protein in *rosy* and DNA in *green*) with the Pab-AGOG + 8-oxodG complex overlaid (in dark cyan). (**D**) Comparison of AP- (*cyan*) and GO-DNA (*rosy*) conformation at the lesion site. (**E**) Overview of Pab-AGOG, h-OGG1 and Mja-OGG2 complexed with GO-containing DNA in the same orientation. HhH motif in *yellow*, GO in *green* and estranged cytosine in *red*. The arrow indicates the conserved loop motif in the three OGG subgroup family.

**Figure 10.**
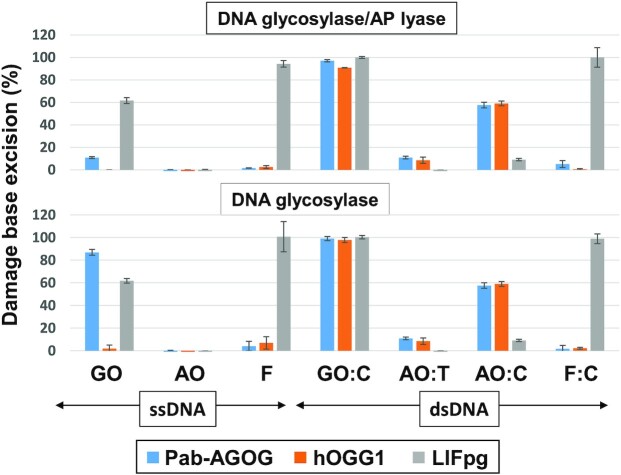
Substrate specificity of Pab-AGOG for several oxidized purines. 20 nM of radiolabeled 24-mer single (ss) and double (ds) stranded DNA containing GO, AO or F (opposite C and/or T) was incubated 15 min at 37°C with 200 nM of Pab-AGOG, hOGG1 and LlFpg as described in Materials and Methods for DNA glycosylase. Mean value of base excision were obtained from at least three independent experiments.

To clarify the mode of recognition of GO in double-stranded DNA prior to the cleavage of its *N*-glycosidic bond by AGOG, we solved the 3D structure of a mutated K142Q-Pab-AGOG, a catalytically defective mutant which has kept its substrate recognition ability (Supplementary Figure S12, Table 3), complexed with a GO-containing 9-mer DNA duplex at 1.25 Å resolution (Pab-AGOG/dsDNA-GO:C, Table 1). In this structure, the protein interacts with the estranged C in the same way as observed for the borohydride-trapped AP lesion with an opposite C (Supplementary Figure S10) while the GO nucleotide is extruded from the DNA helix and stabilized inside the enzyme active site in an *anti*-conformation similar to that previously observed for the OGG1 and OGG2 enzymes (Figure 9A,B) (24,54). The space in the DNA double-helix vacated by the flipped GO is partially filled by Q53 and R93 as seen in the crystal structures of the trapped Pab-AGOG-dsDNA-C (Figure 8C). Compare to the 8-oxodG soaked structure (Table 1, Figure 4), two major differences are seen in the GO binding site (Figure 9C). First, the O8 atom of the GO-containing DNA is no longer contacted by W62 and second, the ribose moiety is flipped and makes an H-bond with D174. This leads to the repositioning of the sidechain of Q142 (K in wild type) towards the GO-sugar moiety. If the side chain of the catalytic lysine could be moved in a similar way upon DNA binding, its ϵ-amino group would be ideally positioned to perform the nucleophilic attack on the C1' of the damaged nucleoside to trigger the excision of GO and the concomitant formation of the transient imino enzyme-DNA intermediate (Figure 5A). Similarly to OGG1 and OGG2, Pab-AGOG does not interact with the 8-oxo-carbonyl of GO but recognizes the N7-H of GO by a strictly conserved glutamine residue (Q24). A proton acceptor at the C8-position and a proton donor at the N7-position of GO are the two chemical determinants that allow distinguishing GO from G. Thus, Q24 is the AGOG residue involved in the specific recognition of GO, equivalent to G42 of OGG1 and the C-terminal K207 of OGG2 (24,53,54). The GO-purine base is sandwiched between the aromatic sidechains of W212 and F146, two residues strictly conserved in AGOGs. The loss of the Pae-AGOG DNA glycosylase/AP lyase activity upon site-directed mutagenesis of W222 to alanine (W212 in Pab-AGOG), K147 to glutamine (K149 in Pab-AGOG) and Q31 to serine (Q24 in Pab-AGOG) is in perfect agreement with our structural model of Pab-AGOG bound to GO-DNA (19). The extensive network of interactions between sidechains of Q24, K149, D174, D208 and atoms N1, N2, 06, N7 of GO highlights the strong selectivity for this lesion and against other oxidized bases such as 8-oxoadenine (AO) or imidazole-ring open purines (FapyG or FapyA). We verified this by examining the ability of Pab-AGOG to excise AO and N7-methyl-FapyG (F) from single- and double-stranded DNA (Supplementary Figure S1, Figure 10). As expected, Pab-AGOG efficiently excises GO from double-stranded DNA, like hOGG1 and LlFpg used as controls. Pab-AGOG and LlFpg are able to excise GO in single-stranded DNA as well, while OGG1 is unable to do so. On the contrary, but as expected based on the X-ray structure of Pab-AGOG bound to a GO-containing DNA duplex, none of the three tested GO-DNA glycosylases can excise AO from single- or double-stranded DNA when it is opposite T (physiological situation). However, as already shown for OGG1, AO opposite C (a non-physiological mispair) is efficiently excised by Pab-AGOG indicating that both these enzymes prefer a C opposite the lesion and that the correct recognition of that mismatch is essential for catalysis (57). As predicted by the 3D structure of Pab-AGOG bound to a GO-containing DNA, the substrate specificity of Pab-AGOG is restricted to GO. The same conclusion has been drawn for Tga-AGOG and TkoAGOG, meaning that these enzymes are unable to excise oxidized pyrimidines such as 5-hydroxy-hydantoin and 5-hydroxy-5-methyl-hydantoin, uracil, OA opposite T, inosine and xanthine (21,23). From this point of view, AGOG and OGG1 have very narrow substrate selectivity for only GO and the AP site, whereas the bacterial GO-DNA glycosylase Fpg – which is structurally unrelated to either OGG protein has a very broad substrate specificity that allows it to remove numerous oxidized purines and pyrimidines from DNA (57–59).

In the Pab-AGOG/dsDNA-GOC structure, the GO lesion is not the only base extruded from the DNA helix. In fact, the thymine upstream of the lesion (T3) is not paired with A7* as expected but rather stacks on the G56 residue of a well-conserved loop between the α4 and α5 helices (Figure 2, Figure 9B, D). In this structure, R176 stacks on W212 and forms a salt-bridge with E59 as observed in the structure of the enzyme bound to GO (R176, W212 and E59 are strictly conserved in AGOGs, Figure 2). Moreover, R176 stabilizes the phosphate group between the two extruded bases (GO and T3), which contrasts with what we observed in the structure Pab-AGOG complexed to the AP site, where no contact between R176 and DNA was present. In the trapped AP site-containing structures, the mainchain of R176 is mobilized, alongside the sidechain of S175, to interact with the C4’-hydroxyl group of the ring-open form of the AP site. Consequently, R176 is a key residue for stabilizing the extruded GO or AP site in the lesion recognition complex, allowing maintaining these lesions in an optimal conformation for catalysis. This can provide a structural explanation for the observation that the alanine substitution of this arginine (R197A) in Tga-AGOG results in a strong decrease of GO excision in single-stranded DNA used as a substrate (22). At the lesion site, the DNA backbone conformation stabilized by R176 is the same as that observed in the 3D structures of OGG1 and OGG2 bound to a GO-containing DNA (indicated by an arrow in Figure 9E). However, the double base flipping on the damaged strand has never been observed for any DNA glycosylases. This unique phenomenon results in a large space being vacated, which could further facilitate acceptance of a purine in an intrahelical conformation opposite GO. In comparison, the absence of the damaged base in the borohydride-trapped Pab-AGOG-AP site complexes frees a much smaller space. Altogether, these structural data could explain why Pab-AGOG is able to more easily accept a purine opposite GO than opposite the AP site.

### Concluding remarks

The objective of this study was to decipher the molecular and structural basis of the recognition of damaged DNA by the hyperthermophilic archaeal GO-DNA glycosylases AGOGs. This objective was achieved by the resolution of several X-ray structures of Pab-AGOG and Tga-AGOG alone or complexed with damaged DNA. By combining biochemical and structural data, the remarkable mechanism of lesion recognition and removal by AGOGs can be summarized as follows:

The strictly conserved catalytic lysine of AGOGs (K142 in Pab-AGOG) is involved in the formation of a transient imino enzyme-DNA covalent intermediate, established between the ϵ-amino group of the lysine and the C1' of the ring-open deoxyribose of GO, which results in GO removal following the cleavage of its *N*-glycosydic bond. The irreversible stabilization of the post-cleavage complex *via* a borohydride-induced linkage to an AP site allowed us to establish the structural basis for recognition of the damaged strand, which is very similar for single- and double-stranded DNA. The enzyme recognizes a few nucleotides on the damaged strand and, importantly, the base opposite the damage and the two phosphates at its 3’ side on the complementary strand.

As known for other DNA glycosylases, AGOGs induce a strong bending of the DNA centered on the damaged nucleotide (GO or AP site), which results in the extrusion of the damaged nucleotide outside the DNA helix. The extruded GO is stabilized in an extrahelical *anti* conformation in the active-site pocket, which leads to its ideal exposure to the nucleophilic attack by K142. Numerous AGOG residues are involved in the recognition of the GO chemical determinants, excluding the recognition of other base lesions. Especially, the N7-proton of GO (distinguishing GO from normal guanine) is contacted by the strictly conserved Q24 residue while the 8-oxo-group of GO is not recognized by the protein, as observed for the GO-DNA glycosylases OGG1 and OGG2.

In the structures of Pab-AGOG bound to GO- and trapped AP site-containing double-stranded DNA with a C opposite the lesion, the space resulting from the flip-out of the damaged nucleotide is filled by three residues wedged in the minor groove of the DNA double helix: Q53, R93 and L94. This intercalated triad first prevents the DNA double helix from locally collapsing and second maintains the opposite C in an intrahelical *anti* conformation. R93 is involved in the recognition of a pyrimidine (C or T) opposite the lesion directly, while Q53 participates in this process indirectly through its interaction with R93 and the damaged strand. These residues jointly ensure the stability of the lesion recognition complex in the double-stranded DNA.

The GO extrusion is associated with an unexpected flip-out of the neighboring base at its 5’ side which mobilizes amino acid residues strictly conserved in AGOGs: G56 in the α4–α5 loop stacks with the second everted base and R176 in the helix-α11 stabilizes the phosphate backbone between the two extruded bases. Such a double base flipping has never been observed for other DNA glycosylases. The resulting large space freed in the double helix allows an intrahelical purine to be accommodated opposite GO without any particular steric hindrance. This phenomenon may provide a structural/mechanistic explanation for the observation that the GO-DNA glycosylase activity of AGOGs is relatively insensitive to the nature of the base opposite the damage in contrast to eukaryotic GO-DNA glycosylases such as hOGG1, which recognizes only a C opposite the damage.

The capacity of archaeal GO-DNA glycosylases such as OGG2 and AGOG to excise GO in single- and double-stranded DNA without a clear selectivity for the base opposite GO remains enigmatic, especially when compared to the well-characterized canonical three-component GO-repair system known in bacteria and higher eukaryotes (*E. coli* Fpg/MutM, MutY and MutT and their human functional counterparts OGG1, MTYH and MTH1, serving the roles of a GO-DNA glycosylase, a A/G specific-DNA glycosylase, and a dGOTPase, respectively—see Introduction). Similarly to OGG2 and AGOG, the bacterial GO-DNA glycosylase (Fpg or MutM) also works on single- and double-stranded DNA and has low selectivity for the base opposite the damage, but MutY can act on the GO:A base pair produced by inaccurate translesion synthesis on a GO:C template, while MutT prevents the incorporation of GO from oxidized dGOTP opposite A. Based on genomic sequence analysis and repair activity in cell extracts, neither MutY nor MutT homologs have been identified/detected in archaea. Even more troubling is the fact that the genetic inactivation of AGOG in the archaeon *Thermococcus kodakarensis* (Tko) results in a similar spectrum of mutations to the isogenic wild-type strain (23). In line with this observation, deletion of the gene encoding Tko-AGOG results in a significant but not total loss of GO-DNA glycosylase activity in the corresponding cell extract. This suggests that, in the absence of AGOG, redundant repair systems prevent GO-induced mutagenesis and ensure the maintenance of genome integrity. In some archea including *Sulfolobus acidocaldarius* and *Sulfolobus solfataricus*, a high-fidelity Y-family DNA polymerase that accurately incorporates a C opposite GO has recently been described as a new mechanism for preventing GO’s mutagenic effect (60). Although archaeal replicative DNA polymerases usually stall at the GO lesion, they can also insert and extend past GO with differing efficiencies, leading to mutagenesis (61). The exchange between replicative DNA polymerase and a more accurate Y-family DNA polymerase (facilitated by the single-strand binding protein, RPA and the replisome scaffold protein, PCNA) allows these organisms to avoid elevated G to T and C to A transversions, compensating for the absence of other GO-repair systems (61,62). It should be noted, however, that there is no DNA polymerase of the Y-family in the archaea from the genus *Thermococcus* and *Pyrococcus* indicating that the GO-repair system is not completely similar in all archaea and further mechanisms might await discovery, especially in AGOG-containing species. The absence of a dGOTPase (a MutT/MTH1 homolog) in archaea could indicate that archaeal DNA polymerases are unable to use dGOTP from the oxidized nucleotide pool as substrate, thus avoiding at least the part of the problem associated with the insertion of A opposite GO during replication. Despite our growing knowledge of the archaeal GO-repair system, some grey areas remain and require further investigation.

## DATA AVAILABILITY

Atomic coordinates and structure factors for the reported crystal structures have been deposited with the Protein Data bank under accession numbers 7OLB, 7OLI, 7OUE, 7OY7, 7P0W, 7P9Z, 7P8L and 7OU3.

Raw gel autoradiographs and experimental data for determination of single-turnover rate constants (Table 2) and dissociation constants (Table 3) are available from the corresponding authors.

## Supplementary Material

gkac932_Supplemental_File
