# Supplementary material for: Structural and functional determinants of the archaeal 8-oxoguanine-DNA glycosylase AGOG for DNA damage recognition and processing
Source: Nucleic Acids Res. 2022 Oct 27;50(19):11072–92. doi: 10.1093/nar/gkac932 (PMC9638937; doi:10.1093/nar/gkac932)
Supplement: gkac932_Supplemental_File [file gkac932_supplemental_file.pdf]

Structural and functional determinants of the archaeal 8-oxoguanine-DNA glycosylase AGOG for DNA damage recognition and processing

Franck Coste, Stéphane Goffinont, Julien Cros, Virginie Gaudon, Martine Guérin, Norbert Garnier, Fabrice Confalonieri, Didier Flament, Marcin Josef Suskiewicz and Bertrand Castaing

Centre de Biophysique Moléculaire, UPR4301 CNRS,

rue Charles Sadron, 45071 Orléans Cedex 02, FRANCE

- Supplementary information –

57-mer GO	CGGAATTCGTCTAGGTTTGAGGT**GO** GACATCGGATCCATGGTACGTCTA GGTCAATGC
57-mer C	GCATTGACCTAGACGTACCATGGA TCCGATGTC**C**ACCTCAAACCTAGA CGAATTCCG
24-mer X	CTGATCGATGAC**X**CCTGACATGAT
24-mer Y	ATCATGTCAGG**Y**GTCATCGATCAG
24-mer F	ACCACGCTAGC**F**AGTCCTAACAAC
24-mer C_F	GTTGTTAGGACT**C**GCTAGCGTGGT
14-mer GO	CTCTTT**GO**TTTCTCG
13-mer GO	CTCTTT**GO**TTTCTC
11-mer GO	CTTT**GO**TTTCTC
9-mer GO	$T_1T_2T_3$**GO**$T_5T_6T_7C_8T_9$
7-mer GO	TT**GO**TTTC
5-mer GO	T**GO**TTT
9-mer Y	AGAAA**Y**AAA

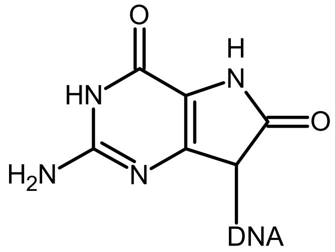

8-oxoG (GO)

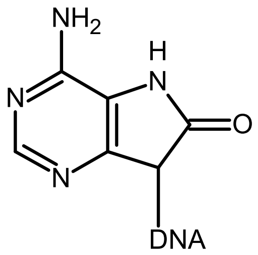

8-oxoA (AO)

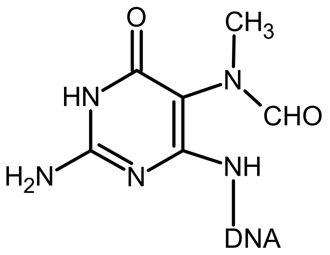

N^7-meFapyG (F)

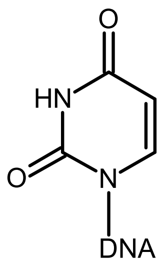

Uracil (U)

Figure S1. Oligonucleotides and structures of damage bases used in this study
X=GO, AO, THF, or U; Y=C, T, G or A; GO=8-oxoguanine (8-oxoG); AO=8-oxoadénine (8-oxoA); F=2-6-diamino-4-hydroxy-5N-methylformamidopyrimidine (N^7-meFapyG).

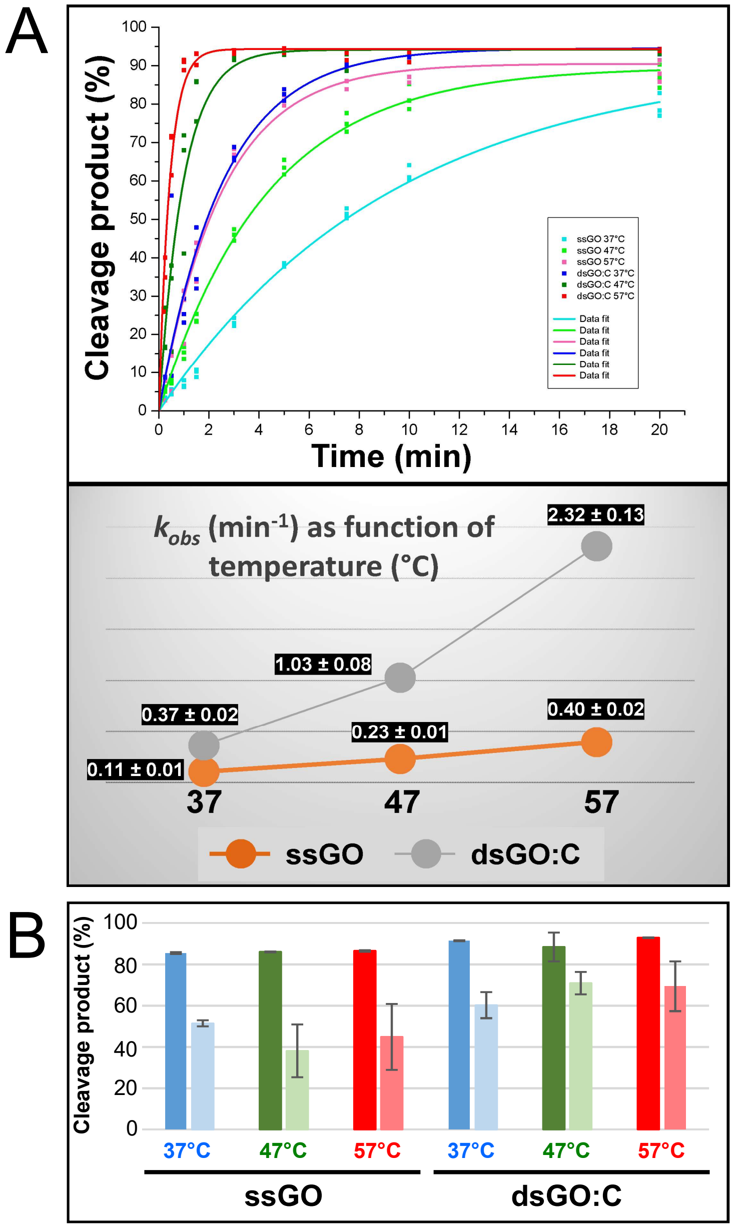

<u>Figure S2</u>: Effect of the temperature on the Pab-AGOG activity

Enzyme assays were performed at 37, 47 and 57°C and analysed as described in *Materials and Methods* using the 57-mer ssGO or dsGO:C as substrate (Fig.S1). (**A**) GO-DNA glycosylase/AP lyase activity under single-turnover conditions (20 nM substrate/200 nM Pab-AGOG). Upper panel: Kinetics curves - for each assay, the final DNA cleavage product is plotted as a function of the incubation time for three independent experiments. Experimental points of each kinetics were fitted as described in *Materials and Methods*. Lower panel: single-turnover rate constants (k_{obs}) extracted from the kinetics curves presented in the upper panel as a function of temperature for ssGO and dsGO:C, as indicated. (**B**) Comparative analysis of GO-DNA glycosylase and GO-DNA glycosylase/AP lyase as a function of temperature. Incubation times were 7, 3, and 2 min for ssGO and 2, 1 and 0,5 min for dsGO at 37, 47 and 57°C in *blue*, *green* and *red*, respectively. Dark and light color bars were for GO-DNA glycosylase and GO-DNA glycosylase/AP lyase activity, respectively. Each bar corresponds to the mean value ± SD obtained for three independent experiments.

Pab-AGOG

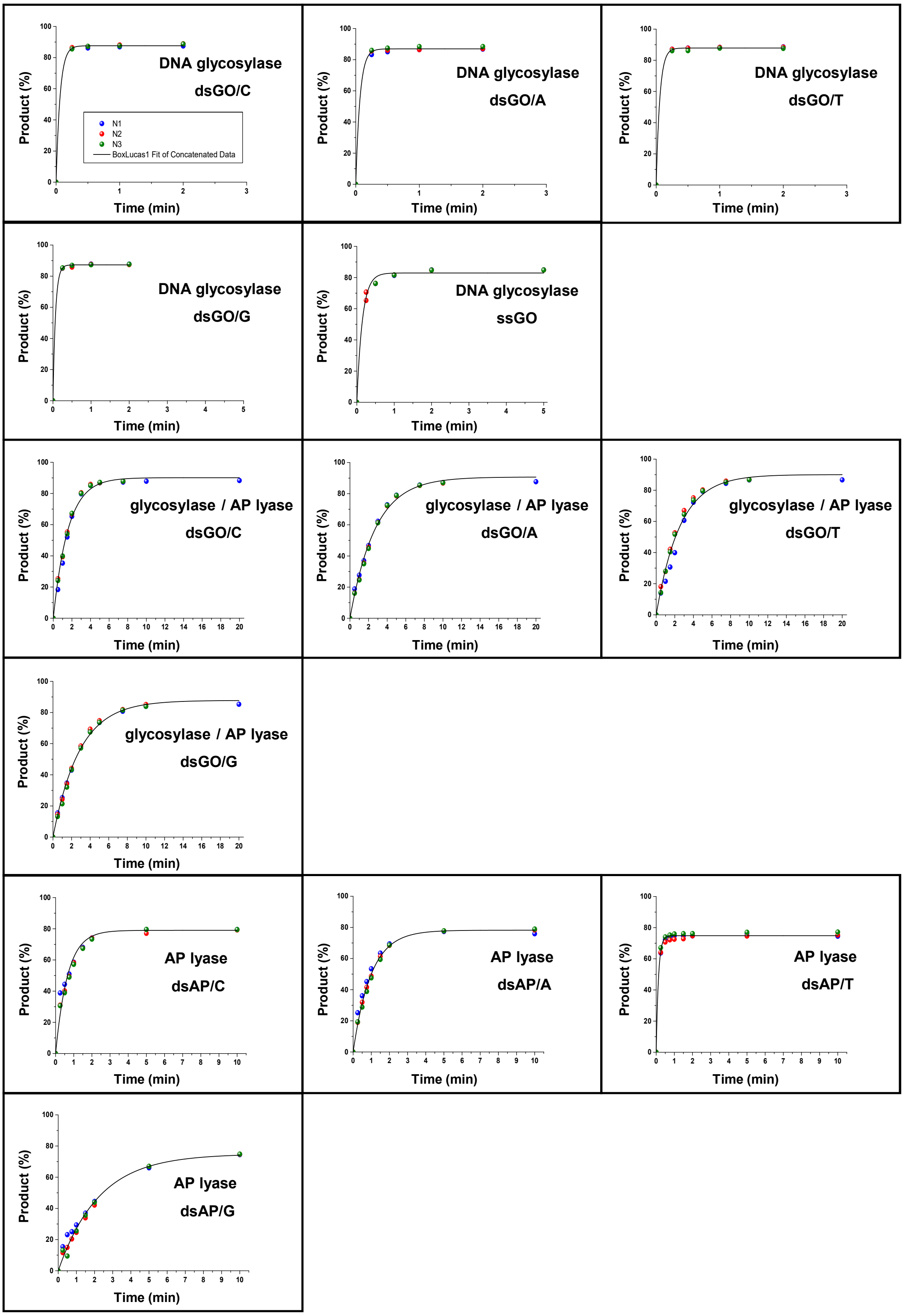

Product (%)
Time (min)
N1
N2
N3
BoxLucas1 Fit of Concatenated Data
DNA glycosylase
dsGO/C
DNA glycosylase
dsGO/A
DNA glycosylase
dsGO/T
DNA glycosylase
dsGO/G
DNA glycosylase
ssGO
glycosylase / AP lyase
dsGO/C
glycosylase / AP lyase
dsGO/A
glycosylase / AP lyase
dsGO/T
glycosylase / AP lyase
dsGO/G
AP lyase
dsAP/C
AP lyase
dsAP/A
AP lyase
dsAP/T
AP lyase
dsAP/G

Tga-AGOG

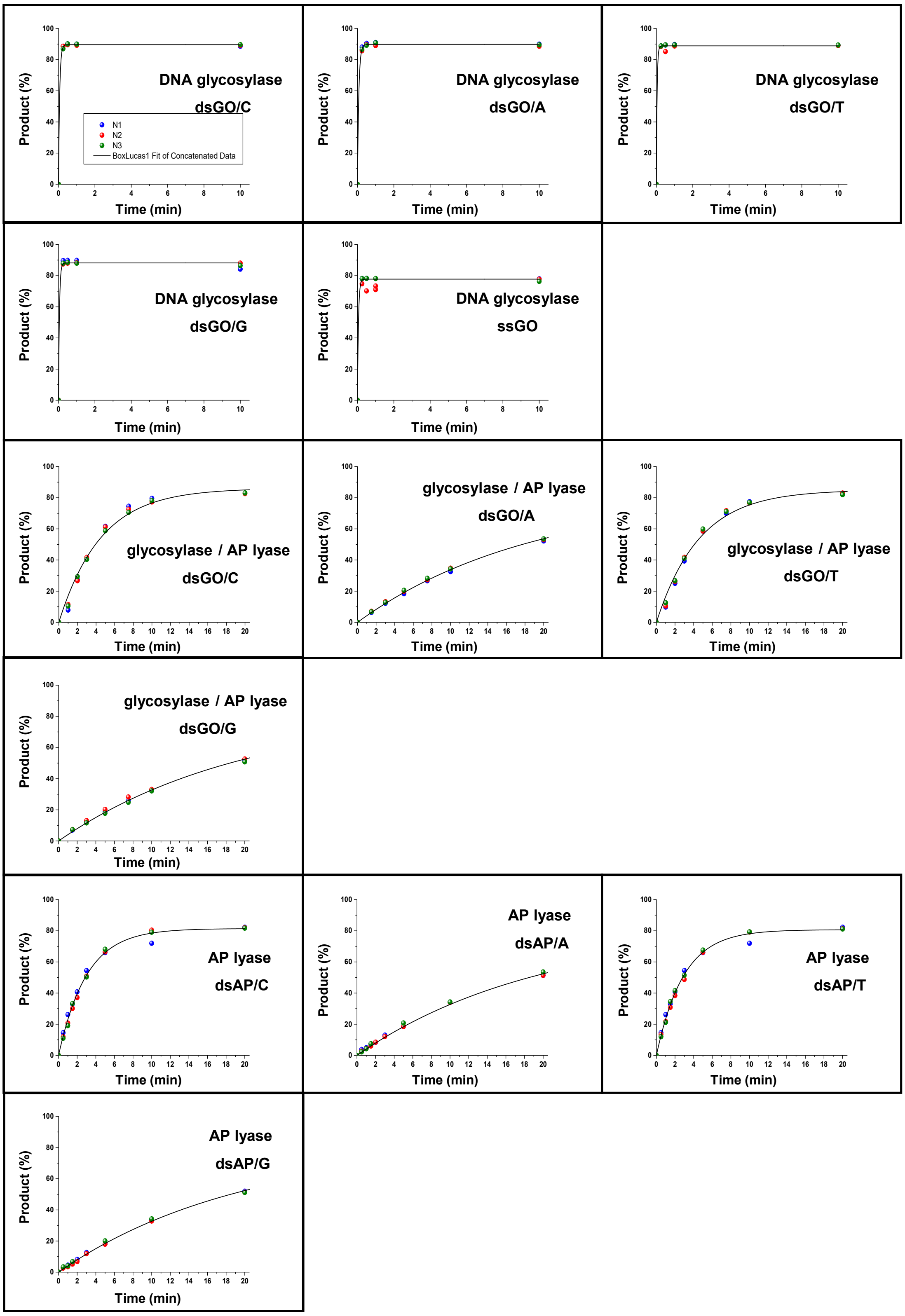

Product (%)
Time (min)
N1
N2
N3
BoxLucas1 Fit of Concatenated Data
DNA glycosylase
dsGO/C
DNA glycosylase
dsGO/A
DNA glycosylase
dsGO/T
DNA glycosylase
dsGO/G
DNA glycosylase
ssGO
glycosylase / AP lyase
dsGO/C
glycosylase / AP lyase
dsGO/A
glycosylase / AP lyase
dsGO/T
glycosylase / AP lyase
dsGO/G
AP lyase
dsAP/C
AP lyase
dsAP/A
AP lyase
dsAP/T
AP lyase
dsAP/G

Figure S3: Kinetics graphs for the determination of single turnover rate constants for Pab- and Tga-AGOG and its modulation by the base opposite the damage

Kinetic assay were performed as described in *Materials & Methods* section with the appropriate substrate as indicated for each graph. Data were for three independent experiments N1, N2 and N3 in *red*, *blue* and *green* points). k_{obs} values presented in table 2 were extracted from these curves.

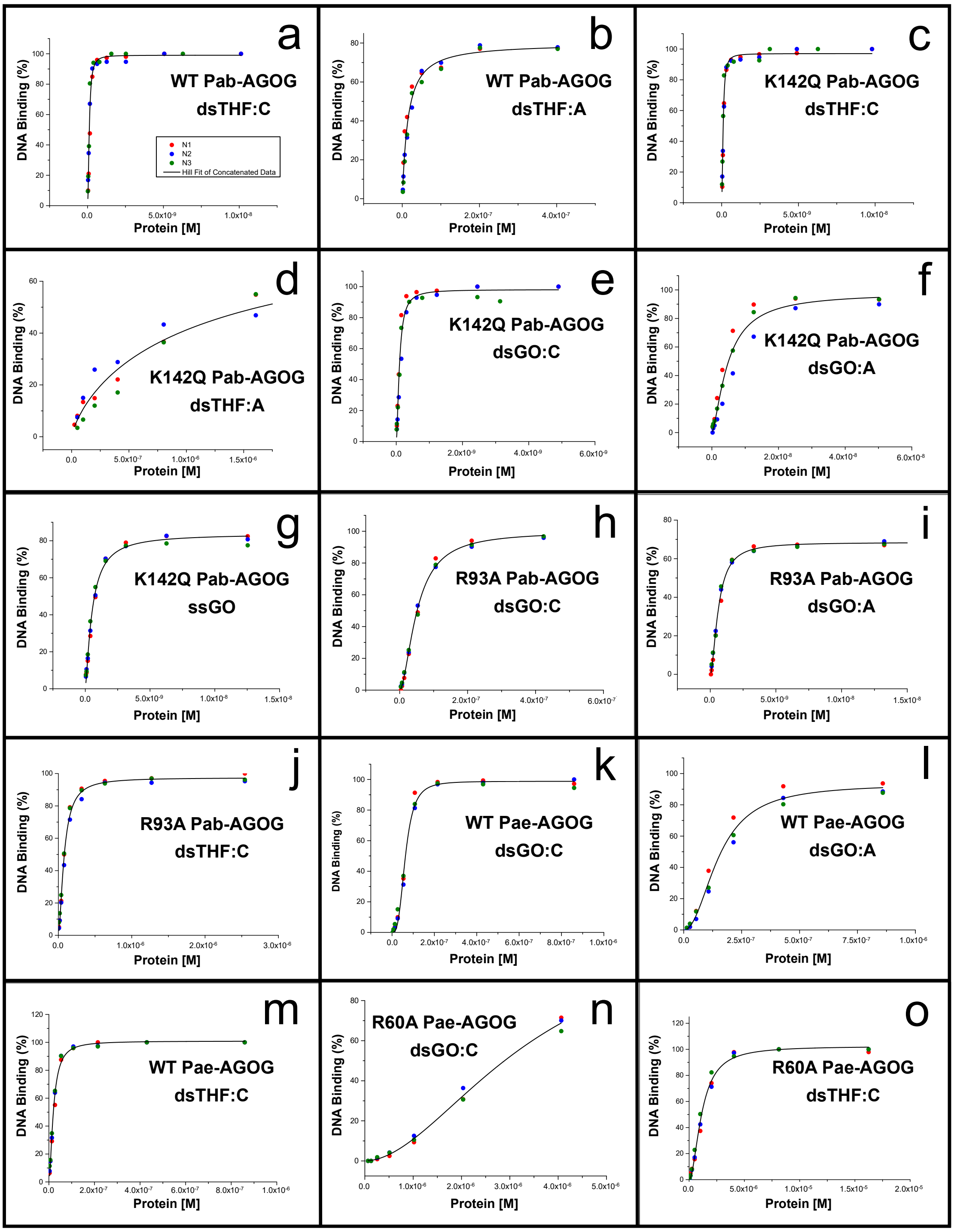

<u>Figure S4</u>: Determination of the dissociation constants between several DNA ligands and wild type or mutants Pab- and Pae-AGOG Binding experiments were performed by EMSA as described in *Materials & Methods* section. Some representative gel autoradiography are presented in Fig.S9. Crude binding data (from three independent experiments N1, N2 and N3 in *red*, *blue* and *green* points, respectively) for each indicated DNA probe (ssGO, dsGO:C, ds GO:A, dsTHF:C or dsTHF:A) and WT (a,b), K142Q (c to g) and R93A (h,i,j) Pab-AGOG and WT (k,l,m) and R60A (n,o) Pae-AGOG were then plotted as a function of protein concentration. Under the conditions used, the protein concentration needed for half-maximal binding is very closed to the dissociation constant K_D. Considering this approximation, we prefer to use the term “apparent dissociation constant” ($K_D app$) whose values are reported in Table 3.

A

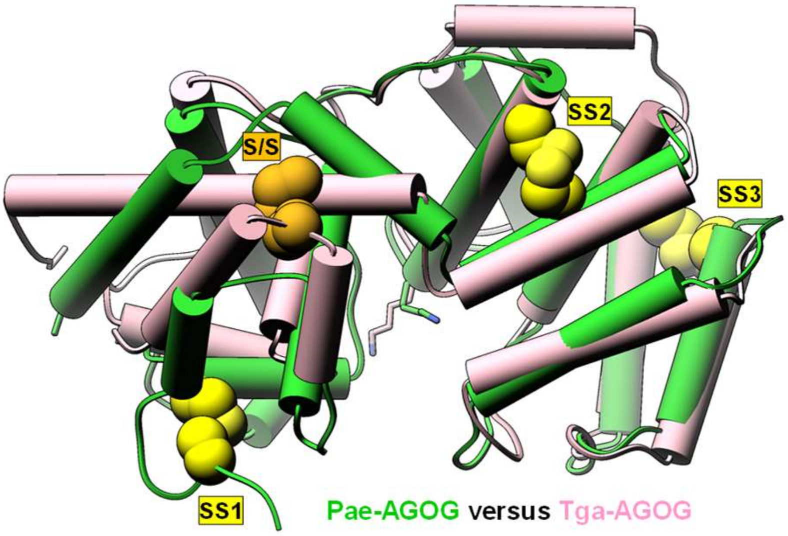

B

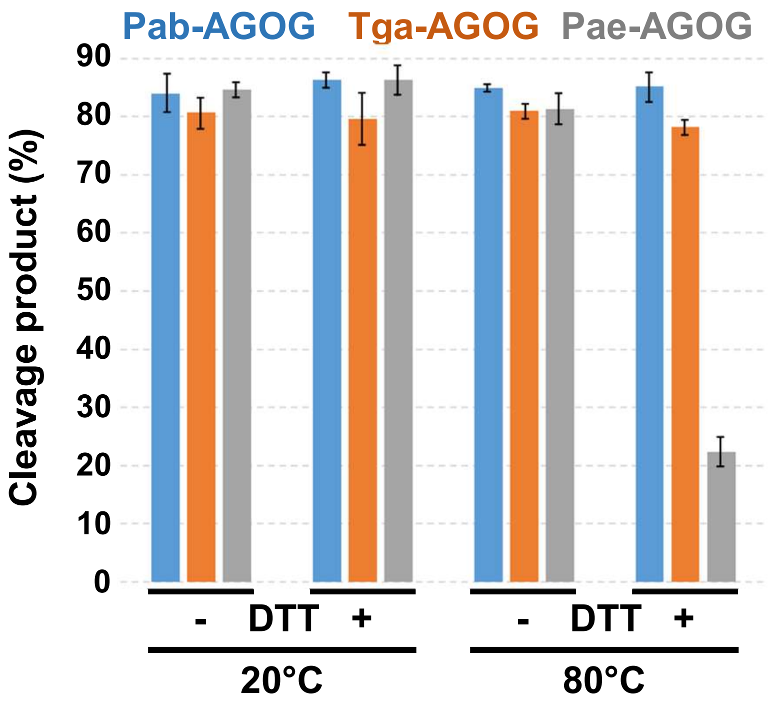

<u>Figure S5</u>. **Potential role of disulfide bridges in Tga- and Pae-AGOG in the maintenance of an active protein structure** (**A**) Superimposition of the crystal structures of Tga-AGOG (in *pink*) and Pae-AGOG (in *green*) highlighting the three disulfide bridges of Pae-AGOG (SS1, SS2 and SS3, in *yellow*) and cysteine residues C32 and C250 (S/S, in *orange*) of Tga-AGOG potentially able to form a disulfide bridge (**B**) Effect of the reducer DTT at 20 and 80°C. After a pre-treatment of AGOGs at indicated temperatures with or without 0.1 M of DTT, proteins were assayed for activity on GO-containing double-stranded DNA at 37°C as described in *Materials and Methods*. The activity expressed in % of DNA cleavage product represents the average of three independent experiments.

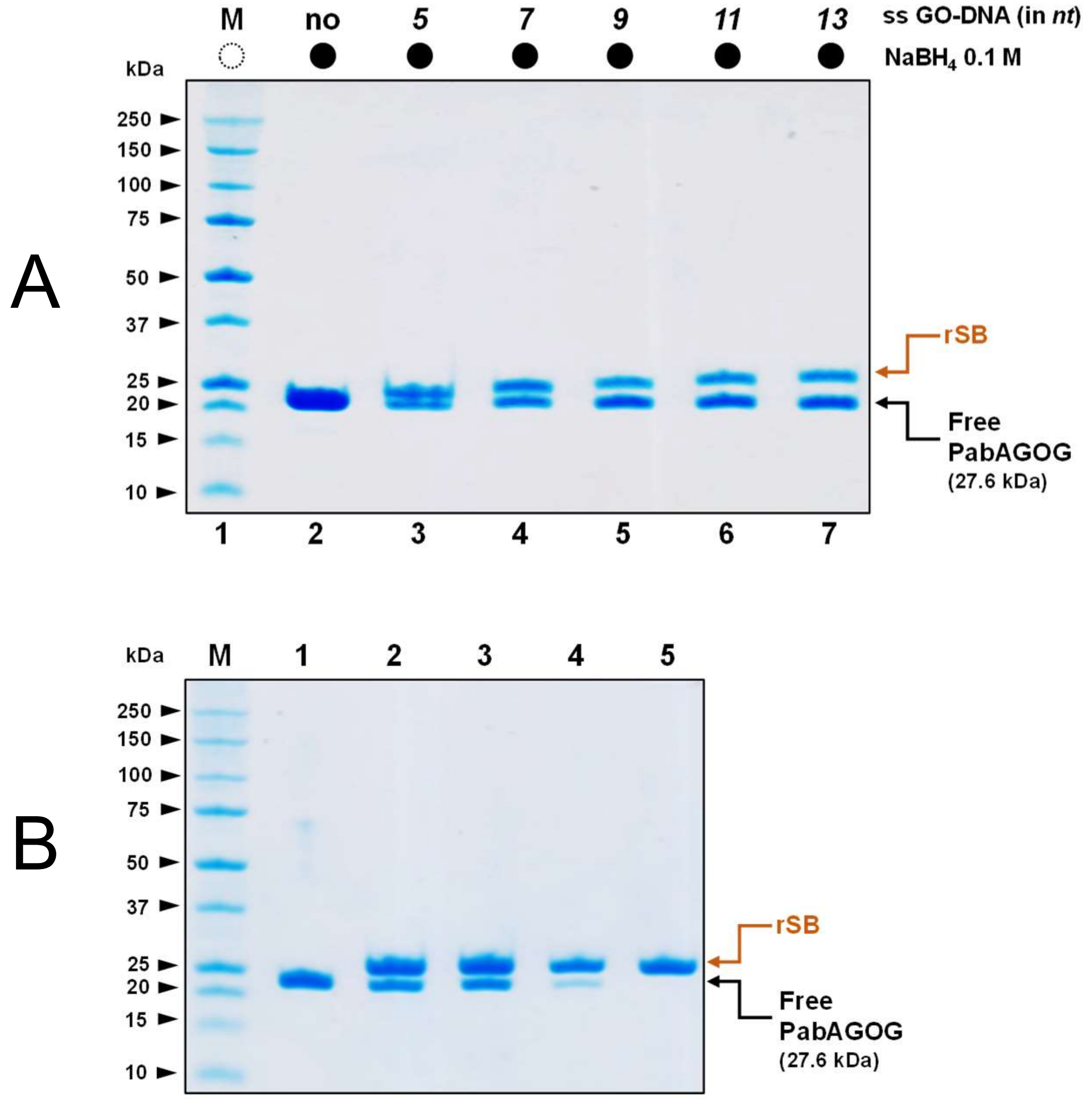

<u>Figure S6</u>: Borohydride-trapping of Pab-AGOG and GO-containing single-stranded DNA.

(**A**) Trapping assays with single stranded DNA of several indicated lengths. (**B**) Purification of the borohydride-trapped complex between Pab-AGOG and GO-containing single-stranded DNA of 9 nucleotides (9-mer GO, Fig.S1): lane 1, purified Pab-AGOG alone; lane 2, trapping mixture before purification; lane 3, elution fraction followed filtration on Superdex 75; lane 4 and 5, homogenous borohydride-trapped complex after 2 successive anion exchanger chromatography on Mono Q.

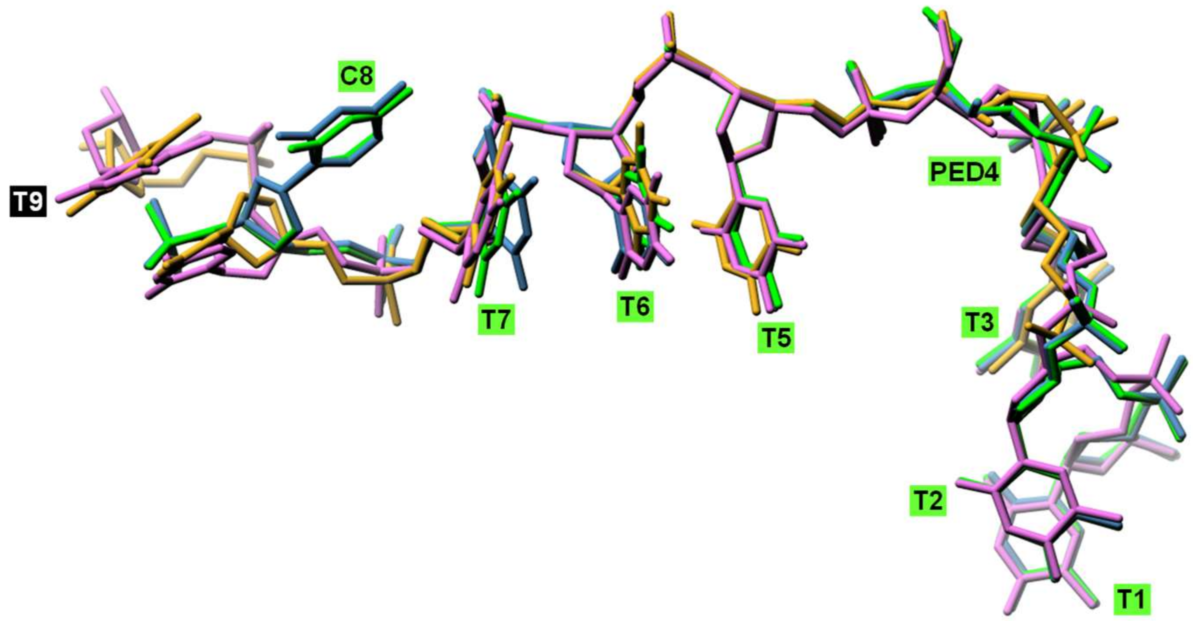

<u>Figure S7</u>. **Superposition of the four single stranded DNA present in the asymetric unit of the Pab-AGOG_ssDNA complex.**

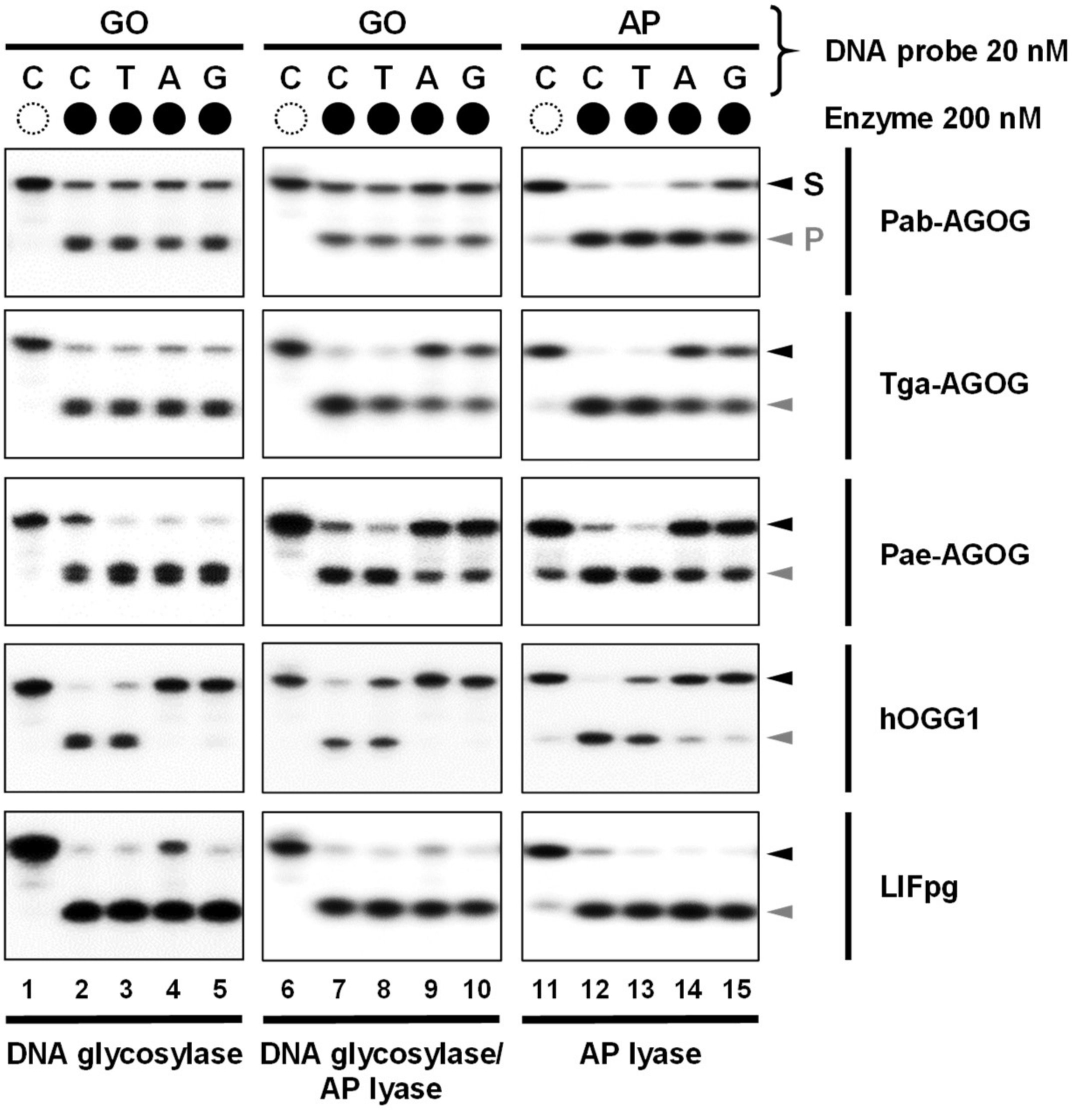

<u>Figure S8</u>. **Comparative analysis of the effect of the base opposite the damage on the activities of several GO-DNA glycosylases under STO conditions.** 20 nM of radiolabeled 24-mer DNA duplex containing GO or AP site opposite C, T, A or G (as indicated) was incubated at 37°C alone (empty dashed circles, GO:C and AP:C, lanes 1 and 6, and 11, respectively) or with 200 nM enzyme (full *black* circles; lanes 2-5, 7-10 and 12-15 for DNA glycosylase, DNA glycosylase/AP lyase and AP lyase activity, respectively). Incubation times were 1 min for DNA glycosylase of all enzymes, and DNA glycosylase/AP lyase of LIFpg and AP lyase of Pab-AGOG and LIFpg, 2 min for DNA glycosylase/AP lyase of Pab-AGOG, 15 and 60 min for ADN glycosylase/AP lyase and AP lyase of Tga-AGOG and hOGG1, respectively. Reaction mixtures were then analyzed by Urea-PAGE as described in *Materials & Methods*. Representative autoradiographs are shown.

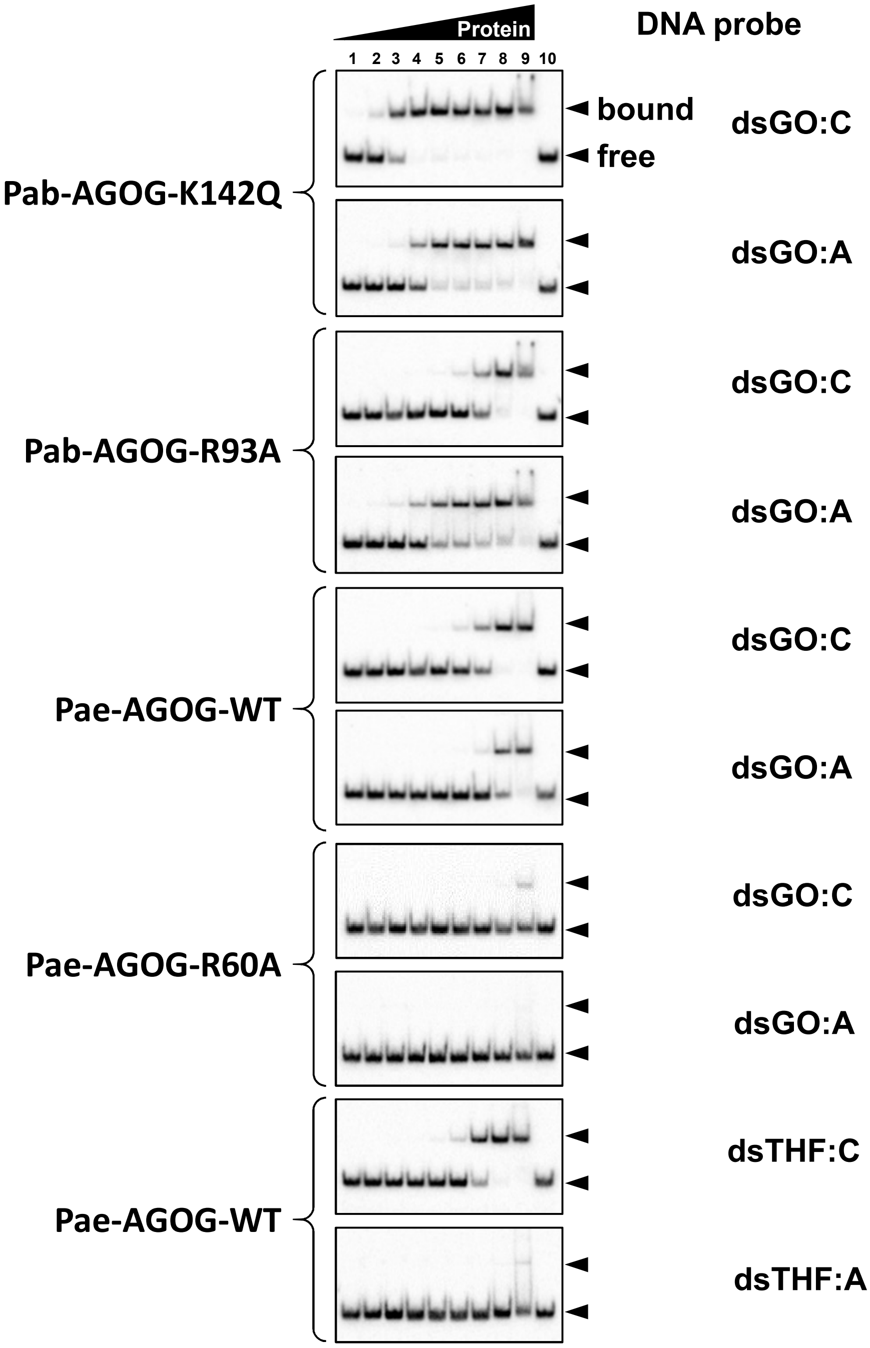

<u>Figure S9</u>. Comparative analysis of the effect of the base opposite the damage on wild type and mutants Pab- and Pae-AGOG DNA binding property. 0.1 nM of radiolabeled 24-mer DNA duplex containing GO or AP site opposite C, T, A or G (as indicated) was incubated at 4°C alone (lane 10), or with 0.013, 0.052, 0.21, 0.83, 3.3, 13, 53, 210, 850 nM of indicated enzyme (lanes 1-9, respectively). Equilibrium mixtures were then analyzed by EMSA. Selected representative autoradiographs are shown. Apparent dissociation constants $K_D app$ were determined by EMSA as described in *Materials & Methods* from at least three independent titration experiments (Fig.S4) and reported in Table 3.

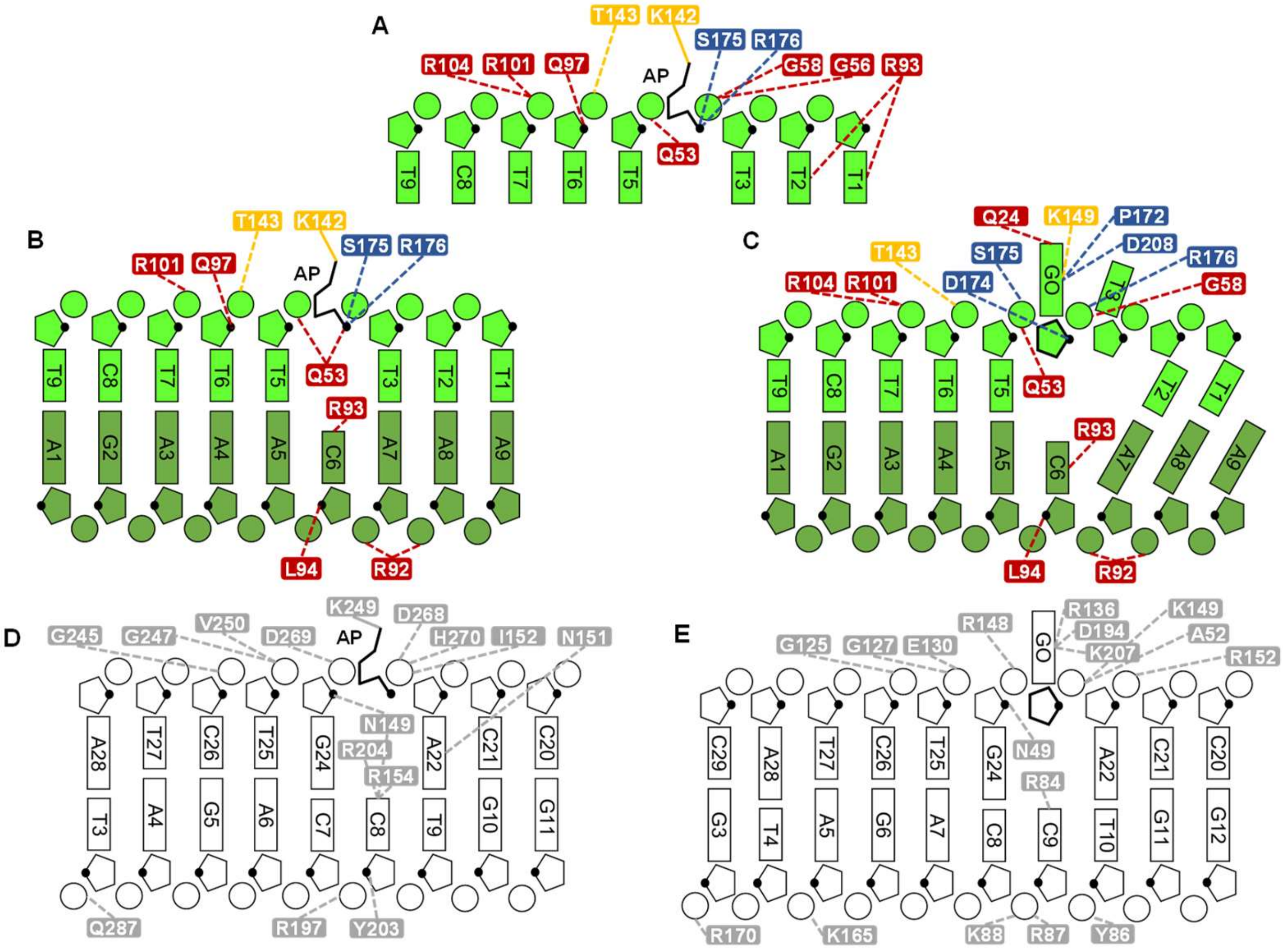

<u>Figure S10</u>. Schematic showing direct interactions between protein and DNA (green circle: phosphate group, green pentagon: ribose, green rectangle: base). (**A**) Pab-AGOG trapped to AP site-containing ssDNA. (**B**) Pab-AGOG trapped to AP site-containing dsDNA with a cytosine opposite AP site (this work). (**C**)) Lesion recognition complex between Pab-AGOG bound to GO-containing dsDNA with a cytosine opposite GO (this work). (**D**) Lesion recognition complex between hOGG1 bound to GO-containing dsDNA with a cytosine opposite GO (PDBid 1HU0). hOGG1 (1HU0). (**E**) Lesion recognition complex between Mja-OGG2 bound to GO-containing dsDNA with a cytosine opposite GO (PDBid 3KNT).

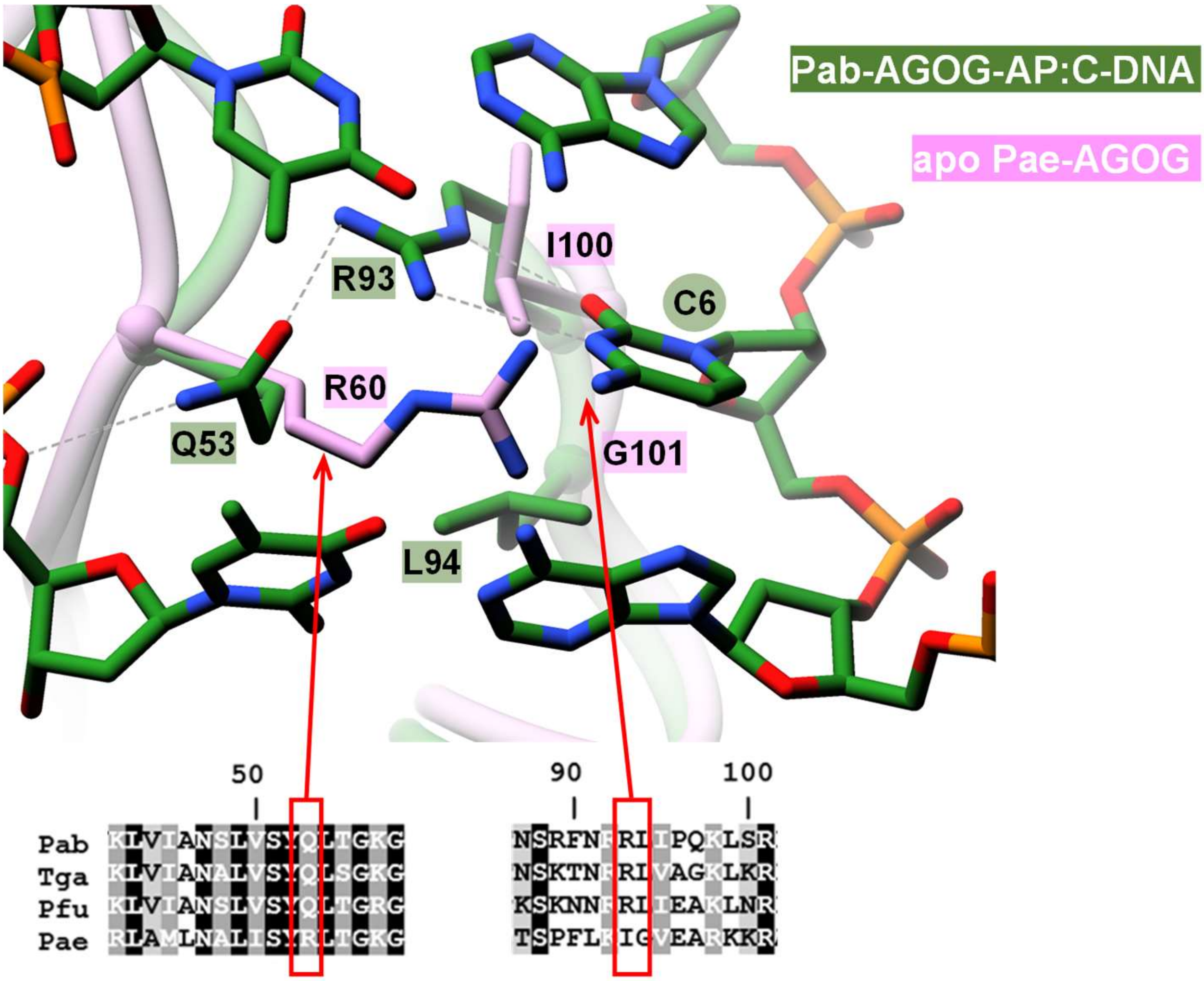

Figure S11. Superposition of the crystal structures of the borohydride-trapped Pab-AGOG-AP-dsDNA complex with a cytosine (C6*) opposite the damage (this work, in *green*) and the apo Pae-AGOG (PDBid 1XQO, in *magenta*).

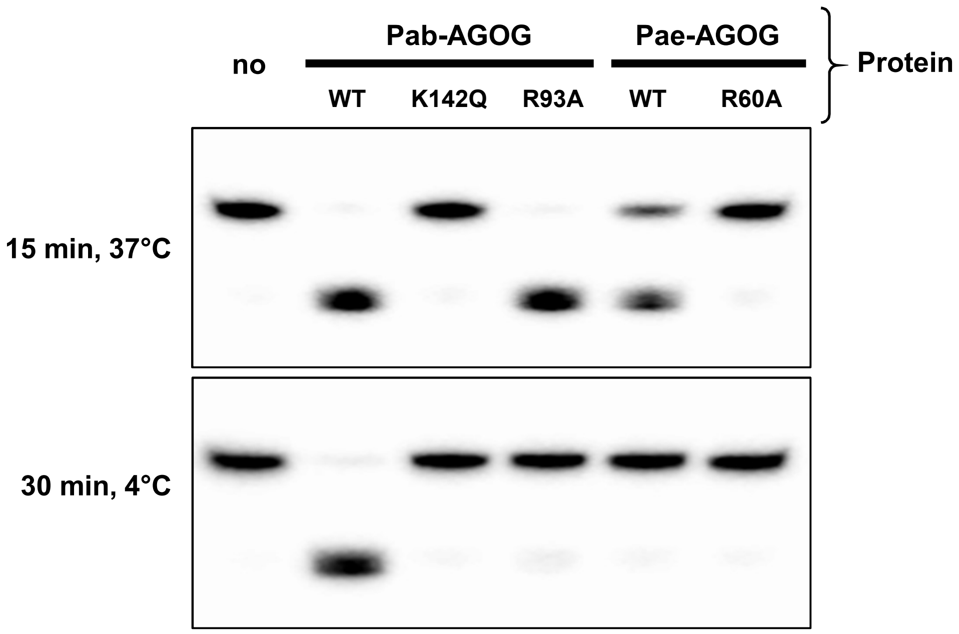

<u>Figure S12</u>. GO-DNA glycosylase activity of wild type and mutants Pab-AGOG and Pae-AGOG

20 nM of radiolabeled 24-mer DNA duplex containing GO opposite C was incubated with 200 nM of indicated protein at 4 or 37°C as indicated. After 15 or 30 min incubation time, reaction mixture were stopped by NaOH and analyzed by Urea-PAGE as described in *Materials and Methods*. Representative autoradiographs are shown.
